# *Albicetus oxymycterus*, a New Generic Name and Redescription of a Basal Physeteroid (Mammalia, Cetacea) from the Miocene of California, and the Evolution of Body Size in Sperm Whales

**DOI:** 10.1371/journal.pone.0135551

**Published:** 2015-12-09

**Authors:** Alexandra T. Boersma, Nicholas D. Pyenson

**Affiliations:** 1 Department of Earth Sciences & Geography, Vassar College, Poughkeepsie, NY, 12604, United States of America; 2 Department of Paleobiology, National Museum of Natural History, Smithsonian Institution, PO Box 37012, Washington, DC, 20013, United States of America; 3 Departments of Mammalogy and Paleontology, Burke Museum of Natural History and Culture, Seattle, WA, 98195, United States of America; New York Institute of Technology College of Osteopathic Medicine, UNITED STATES

## Abstract

Living sperm whales are represented by only three species (*Physeter macrocephalus*, *Kogia breviceps* and *Kogia sima*), but their fossil record provides evidence of an ecologically diverse array of different forms, including morphologies and body sizes without analog among living physeteroids. Here we provide a redescription of *Ontocetus oxymycterus*, a large but incomplete fossil sperm whale specimen from the middle Miocene Monterey Formation of California, described by Remington Kellogg in 1925. The type specimen consists of a partial rostrum, both mandibles, an isolated upper rostrum fragment, and incomplete tooth fragments. Although incomplete, these remains exhibit characteristics that, when combined, set it apart morphologically from all other known physeteroids (e.g., a closed mesorostral groove, and the retention of enameled tooth crowns). Kellogg originally placed this species in the genus *Ontocetus*, a enigmatic tooth taxon reported from the 19^th^ century, based on similarities between the type specimen *Ontocetus emmonsi* and the conspicuously large lower dentition of *Ontocetus oxymycterus*. However, the type of the genus *Ontocetus* is now known to represent a walrus tusk (belonging to fossil Odobenidae) instead of a cetacean tooth. Thus, we assign this species to the new genus *Albicetus*, creating the new combination of *Albicetus oxymycterus*, gen. nov. We provide new morphological observations of the type specimen, including a 3D model. We also calculate a total length of approximately 6 m in life, using cranial proxies of body size for physeteroids. Lastly, a phylogenetic analysis of *Albicetus oxymycterus* with other fossil and living Physeteroidea resolves its position as a stem physeteroid, implying that large body size and robust dentition in physeteroids evolved multiple times and in distantly related lineages.

## Introduction

Living sperm whales are represented by three species (*Physeter macrocephalus* Linneaus [1] *Kogia breviceps* Blainville [2] and *Kogia sima* Owen [3]) that are found throughout the world’s oceans. The species *Physeter macrocephalus* is the largest living toothed whale, with adults reaching approximately 18 m in length [4, 5]. *Physeter macrocephalus* also ranks among the deepest diving marine mammals [6], lives in complex social groups, and remains relatively abundant despite prolonged and geographically widespread eras of whaling [7]. *Physeter* is the sister taxon to the living genus *Kogia*, represented by the much smaller pygmy sperm whales (*Kogia breviceps*), and the dwarf sperm whales (*Kogia sima*), which reach up to about 2.7 m and 3.5 m in length respectively [8]. Together, *Physeter* and *Kogia* form the crown group Physeteroidea (sensu Velez-Juarbe et al. [9]), which can be distinguished from all other toothed whales (Odontoceti) by major morphological traits in the skull, including: a severe left (or sinistral) asymmetry of the bones in the dorsal narial region of the cranium, resulting in the loss of one or both of the nasals; and the presence of a large supracranial basin to house the spermaceti organ [4, 10–11].

Physeteroidea is consistently recovered as the first branching lineage of extant Odontoceti in molecular, morphological, and combined phylogenetic analyses (e.g., [12–15]). Some initial analyses of cetacean molecular data in the 1990s grouped sperm whales with baleen whales, to the exclusion of all other odontocetes, but this result is now regarded as spurious by all subsequent systematists and a case study in incorrect phylogenetic rooting [16–17]. Physeteroids are also among the oldest lineages of crown Cetacea, with the oldest putative fossil sperm whale, *Ferecetotherium kelloggi* Mchelidze [18], reported from the late Oligocene of Azerbaijan [4, 18–19]. The Neogene fossil record of this group is taxonomically diverse, with multiple species of physeteroids found in contemporaneous fossil cetacean assemblages (e.g. [20]), representing a range of body sizes between *Kogia* spp. and *Physeter*. Many fossil Physeteroidea retain upper teeth and enamel apices [21–24] while taxa more closely related to *Physeter* have reduced or vestigial upper teeth without enamel, such as *Aulophyseter morricei* Kellogg [3, 25]. Many fossil physeteroid taxa have been described on the basis of questionably diagnostic isolated fragments of specimens, including teeth and vertebrae, and are therefore considered incertae sedis [26–27].

**Fig 1:**
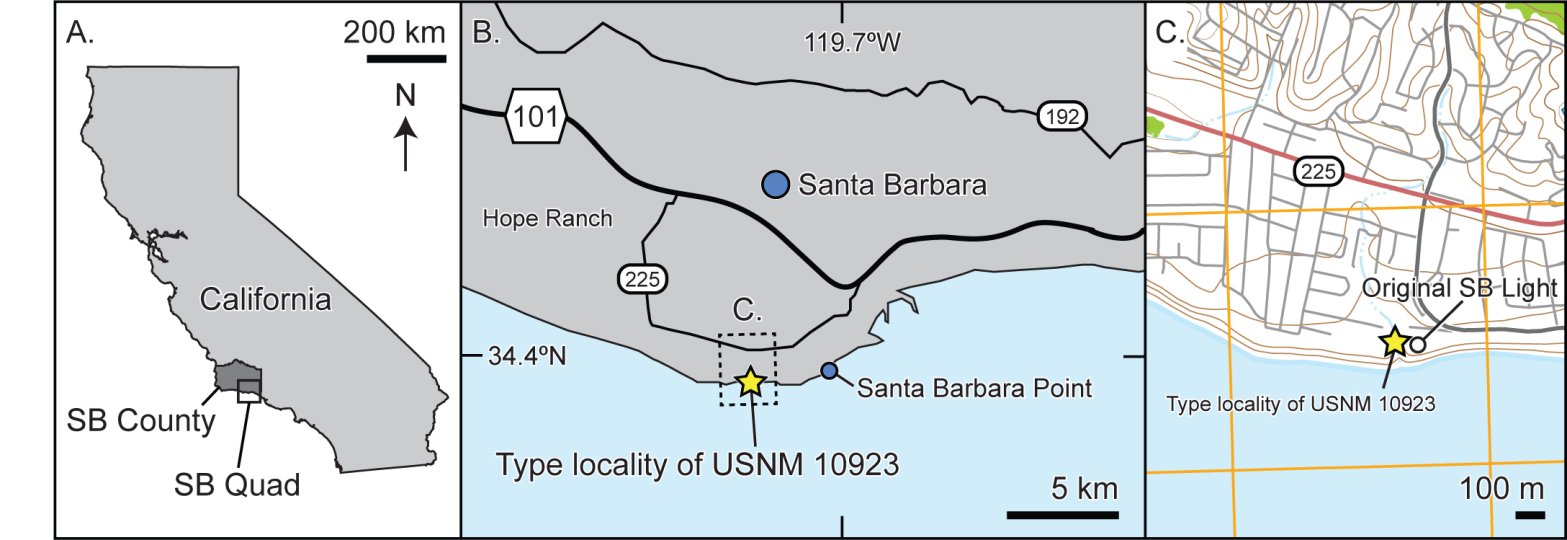
Map of type locality for *Albicetus oxymycterus* (USNM 10923). A, a map of the state of California, showing Santa Barbara County and a box indicating the 2015 United States Geological Survey 7.5 minute topographic map for Santa Barbara Quadrangle, used for B and C. B, a general outline of the vicinity, showing the city of Santa Barbara, with major highways, Santa Barbara Point, and the type locality. Dashed box indicates the area in C, a map of the coast around the type locality, with the location of the original Santa Barbara lighthouse, using a modified basemap from the USGS topographic map (available at http://usgs.gov). Isobars are 50 meters, and cartographic north for all panels points to the top.

**Fig 2:**
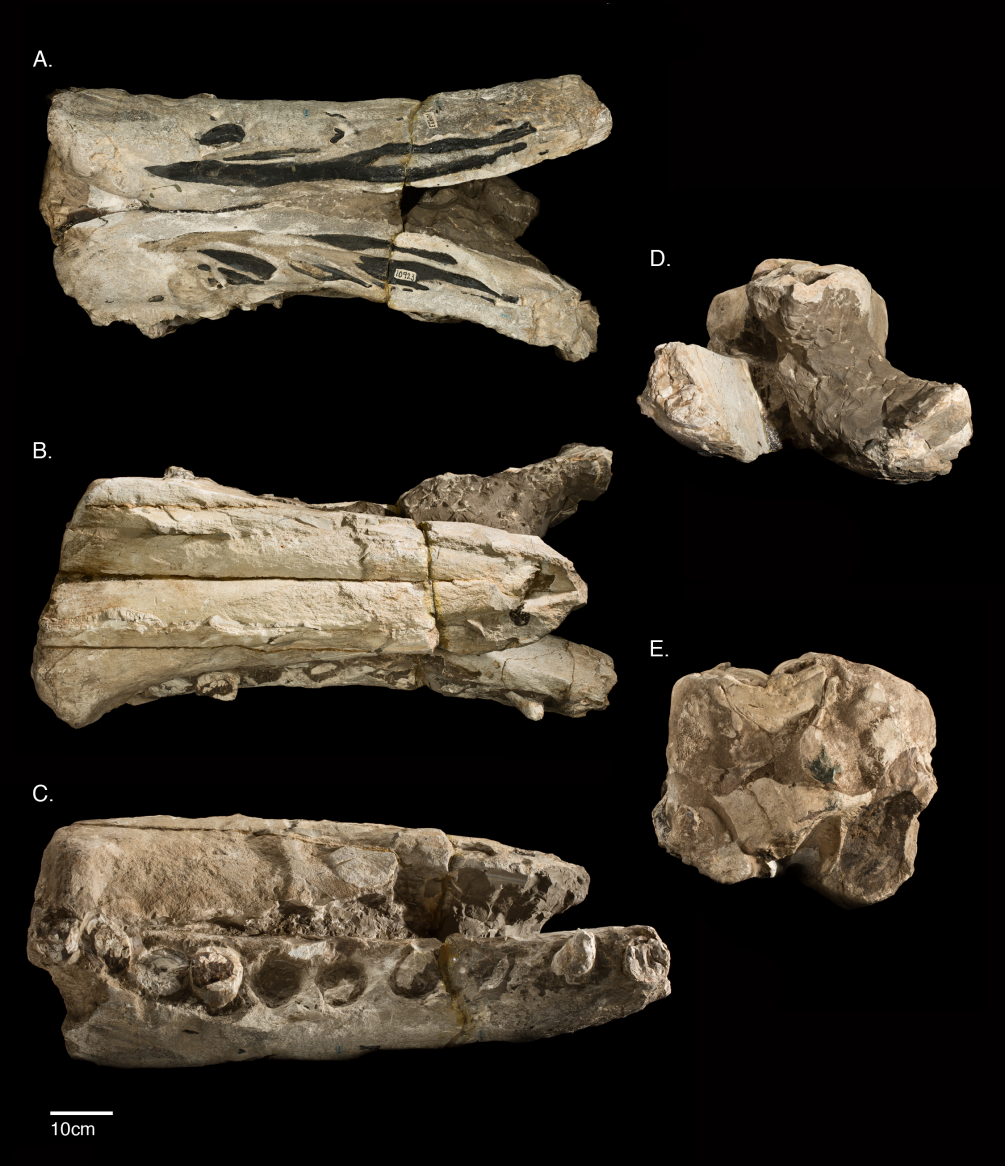
The rostrum and mandibles of *Albicetus oxymycterus* (USNM 10923). A, the rostrum and mandibles together in ventral view, B in dorsal view, C in right lateral view, D in anterior view, and E in posterior view.

**Fig 3:**
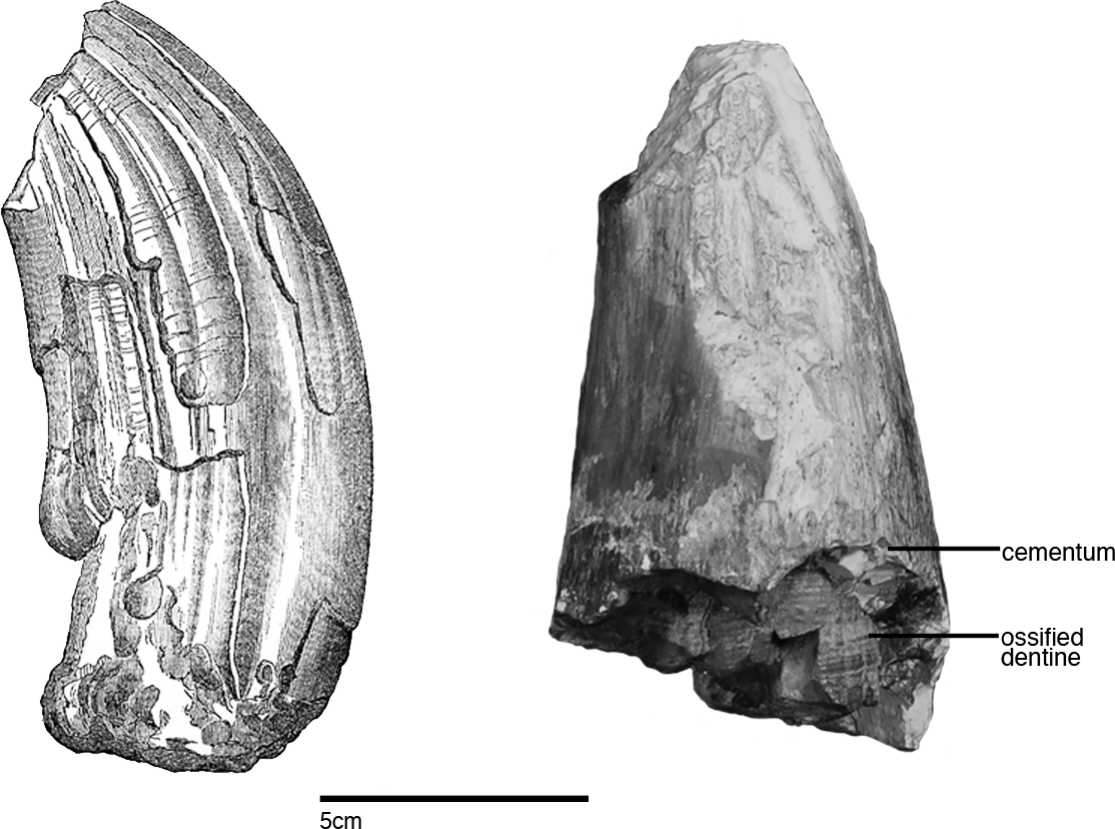
Comparison of type specimen *Ontocetus emmonsi* with a tooth from type specimen *Albicetus oxymycterus*. Illustration by Joseph Leidy of the type specimen of *Ontocetus emmonsi* (USNM 329064) (left), published by Emmons [40], who described the material as originating from “the older Miocene of White River [which] has furnished remarkable animals remains …[among] these remains ruminants are particularly worthy of note.… The Cetacean, Fig 187 (2), is a remarkable form of tooth for this family—having a resemblance to the canine of the Hippopotamus." This illustration is contrasted with an isolated tooth from *Albicetus oxymycterus* (USNM 10923) (right), with the layers of the dentition labeled. Scale bar measures 5 cm. A 3D model of the type specimen *Ontocetus emmonsi* is now available for viewing and download on the Smithsonian X 3D website (http://3d.si.edu).

This paper aims to redescribe *Ontocetus oxymycterus* Kellogg [28], a large fossil sperm whale described from the Monterey Formation of Santa Barbara County, California, U.S.A (Fig 1). The type specimen consists of an incomplete rostrum, and isolated fragment of the upper rostrum, both mandibles with some large dental roots, and several isolated, but associated incomplete teeth (Fig 2). Kellogg [28] tentatively referred the species to the genus *Ontocetus*, based primarily on its large, conspicuous teeth, which he thought resembled *Ontocetus emmonsi* Leidy [29], a tooth taxon from the Neogene of North Carolina [30] (Fig 3).

### Taxonomic history

The type specimen of *Ontocetus emmonsi* (now USNM 329064) is represented by a single, large, laterally compressed, tusk-like tooth, which was originally deposited in 1860 at Williams College, Williamstown, Massachusetts, and was first reported in a textbook illustration by Emmons [31]. The same year, Leidy [29] formally designated this specimen as the holotype of *Ontocetus emmonsi* (Fig 3). Although the exact provenance of this tooth is unknown, Leidy [30] mentioned “miocene [sic] deposits of North Carolina,” likely communicated to him by Emmons. The type specimen (USNM 329064) is incomplete, but it exhibits long, thick, and cambered striae of dentine, with a subrectangular outline in transverse section (Fig 3). These features are utterly absent from all known physeteroid teeth, which are typically conical towards the apex, cylindrical in transverse section, and gibbous towards the root.

With these traits in mind, Leidy [30] later suggested that *Ontocetus emmonsi* represented either a “cetacean like the Sperm Whale,” or “perhaps to a walrus-like animal.” Brandt [32] similarly designated the type species as a toothed whale, but concurred with Leidy’s alternative identification (see Spamer et al. [33], for a detailed bibliographic history). Although Matsumoto [34], Shikama et al. [35] and Okazaki [36] reported cetacean fossil material from Japan belonging to this species, all other recent authors have thoroughly discounted any cetacean affinities to the type specimen *Ontocetus emmonsi*. Ray [37], Kohno and Ray [38], Boessenecker and Churchill [39], and Churchill et al. [40], among others, have all confirmed the odobenid identity of *Ontocetus emmonsi*. The genus *Ontocetus* remains a valid stem odobenid taxon, with additional species yet to be named and described [40].

Following this realization, Kohno and Ray [38] provisionally regarded *Ontocetus oxymycterus* as belonging to the genus *Scaldicetus* de Bus [41], a cosmopolitan fossil physeteroid distinguished primarily by enamel capped and gibbous teeth [24]. However, this recommendation is unsatisfactory, as the genus *Scaldicetus* is a form taxon representing a number of isolated teeth [24] that may or may not belong to a single physeteroid taxon [22–23]. Moreover, the type specimen of *Ontocetus oxymycterus* exhibits a number of diagnostic morphological characters, including proportionately large dentition (relative to rostral size), retention of tooth enamel, and a closed mesorostral groove, which combined distinguish it from all other described physeteroid genera. Because the type species of *Ontocetus* is not a cetacean, we propose a new generic name, *Albicetus*, for the species of *Ontocetus oxymycterus* [28], and provide a redescription of the type specimen, along with body size estimates and a phylogenetic analysis to resolve its relationship and evolutionary context among Physeteroidea.

## Materials and Methods

For specimens observed, see S1 Table.

Anatomical terminology was taken from Mead and Fordyce [42], with modifications from Fitzgerald [24] and Velez-Juarbe et al. [9].

No permits were required for the described study because the material was collected from an undocumented locality between 1879 and 1909.

### 1. 3D digitization procedures

**Fig 4:**
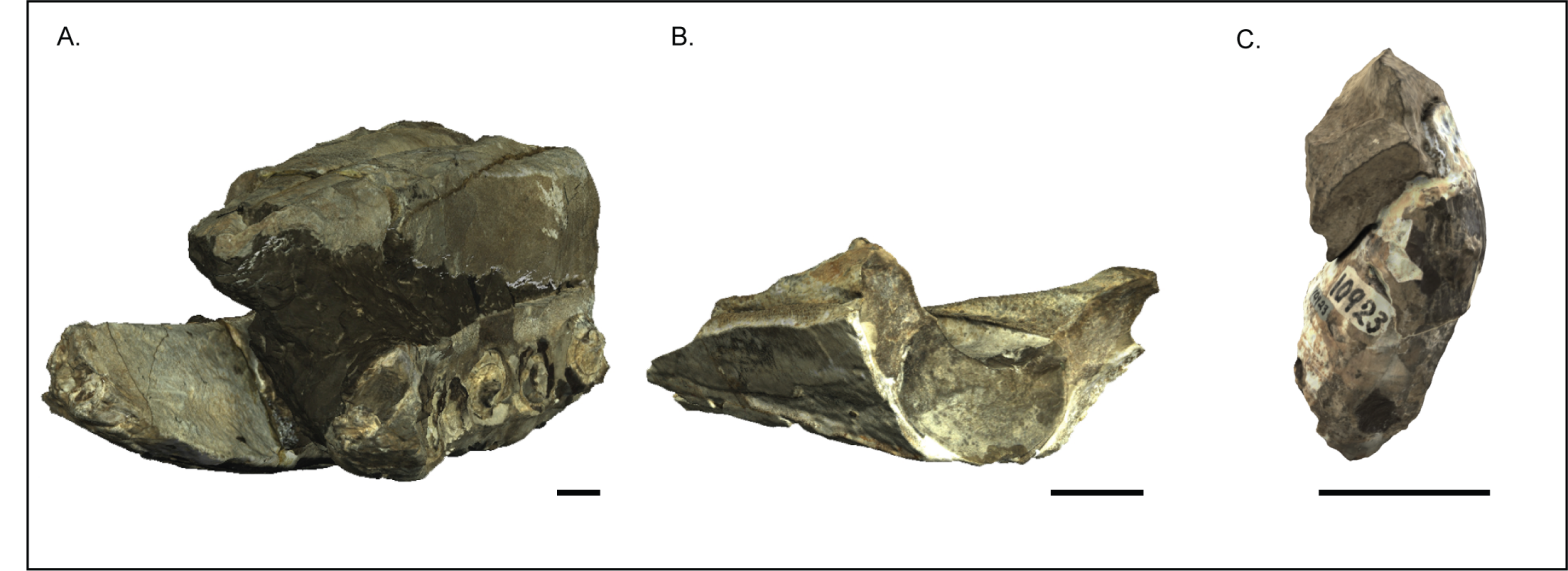
Structured light 3D model of *Albicetus oxymycterus* (USNM 10923). A, rostrum and mandibles in oblique anterior-left lateral view, B, isolated upper rostrum fragment in posterior view, and C, isolated tooth fragment with enamel cap. Scale bars measure 5 cm. The 3D model is accessible through the Smithsonian X 3D website (http://3d.si.edu).

The rostrum and mandibles section that comprise the main parts of USNM 10923 measure almost 1 m in the longest dimension and weigh over 100 kg, requiring at least four adults to manipulate and move it (Fig 2). These daunting logistics for morphological comparisons make it challenging to study all the standard anatomical views and any oblique angles. Thus, we collected three-dimensional datasets of its surface topology using an Artec Eva structured light scanner (Artec Group, Palo Alto, California), scanning at 8 frames per second. These datasets were then compiled in Geomagic version 2012 (3D Systems, Rock Hill, South Carolina) and the free software Meshlab to render a 3D surface model of the type specimen, including the main rostrum and mandibles section, as well as the separated isolated upper rostrum fragment, and an isolated tooth fragment with tooth enamel (Fig 4). The size and density of the bone and matrix for the specimen block containing the rostrum and mandibles exceeds the abilities of industrial x-ray computed tomography facilities, and thus a 3D surface model permitted us to observe the specimen from angles that would have otherwise been impossible. We did however, CT scan a small isolated tooth fragment exposing part of the enamel cap in a Nikon Metrology 225kV microfocus CT scanning system at Chesapeake Testing (Belcamp, Maryland). The 3D models are available for manipulation, visualization, sharing and download through the public data repository Zenodo https://zenodo.org/record/23029 <https://zenodo.org/deposit/43082/> (doi:10.5281/ zenodo.23029) as well as through the Smithsonian X 3D website (http://3d.si.edu).

### 2. Body size estimates

We used two methods to estimate the condylobasal length and total length of *Albicetus*. Since the type specimen of *Albicetus* lacked other skull measurements needed for partial least squares (PLS) mulivariate regression equations, both approaches used a bivariate ordinary least squares (OLS) linear regression with antorbital notch width as a single body length proxy [43]. The antorbital notch was not preserved on the left side of the rostrum, and thus we collected this measurement by measuring from the right side of the specimen, and then doubled it for a minimum estimate of the antorbital notch width (also often referred to as width at rostrum base) (See S2 Fig for a visual guide to measurements). The antorbital notch is not preserved in its entirety in the type specimen, and the measurement therefore likely corresponds to a level just slightly anterior to the antorbital notch, though we refer to it as antorbital notch width in this paper.

The first method used a bivariate OLS linear regression to plot condylobasal length for 36 specimens of extant and fossil physeteroids, including *Physeter macrocephalus*, *Kogia breviceps*, *Kogia sima*, *Aulophyseter morricei* and *Orycterocetus crocodilinus* Cope [44] (see S4 Table of condylobasal lengths). The trend line for this regression produced an equation to estimate the condylobasal length of *Albicetus*, based on the antorbital notch width of USNM 10923. We then calculated the total length of *Albicetus* using a bivariate OLS linear regression by plotting total length against condylobasal length for specimens with associated total length data (only *Kogia* and *Physeter* specimens). The equation from this latter regression, along with the estimated condylobasal length of *Albicetus* from the first regression, provided the basis for calculating an estimated total length for *Albicetus*. The second method was similar to the first, except that we subtracted rostrum length from condylobasal length, and condylobasal length from total length, respectively, as the y-axis values, to account for varying rostrum lengths in specimens (see a similar approach in Lambert et al. [5]).

### 3. Phylogenetic analysis

To assess the phylogenetic relationships of *Albicetus*, we undertook a phylogenetic analysis using a matrix of 42 morphological characters, edited by Velez-Juarbe et al. [9] from Lambert et al.’s [5] original description of *Livyatan melvillei* Lambert et al. [5]. We added one additional character to this matrix, which allowed us to code for the mesorostral groove being open (ancestral state), partially open at the level of the antorbital notches (as in the case of *Brygmophyseter* Kimura et al. [4], *Aulophyseter*, and *Scaphokogia* Muizon [45]), or roofed over at the level of the antorbital notches as in *Albicetus*, with the premaxillae angled downward into the midline, creating a trough down the middle of the rostrum (derived state). The addition of *Albicetus* made a total of 21 operational taxonomic units in the analysis. The Miocene physeteroids from Patagonia, including *Idiorophus patagonicus*, *Diaphorocetus poucheti* and *'Aulophyseter' rionegrensis*, were not added to the matrix. These taxa are fragmentary and poorly described, and thus would not have provided reliable characters to add to the matrix. The cladistic search was performed on PAUP* [46], using all characters as unordered. First, we performed a heuristic search using the tree bisection-reconnection (TBR) algorithm. We then conducted subsequent statistical support analyses by searching for successively longer trees to calculate decay indices and 100 bootstrap replicates. The complete matrix and description of character states (S2 and S3 Tables) are available in the Supplementary Information material.

### 4. Nomenclature acts

The electronic edition of this article conforms to the requirements of the amended International Code of Zoological Nomenclature, and hence the new names contained herein are available under that Code from the electronic edition of this article. This published work and the nomenclatural acts it contains have been registered in ZooBank, the online registration system for the ICZN. The ZooBank LSIDs (Life Science Identifiers) can be resolved and the associated information viewed through any standard web browser by appending the LSID to the prefix “http://zoobank.org/”. The LSID for this publication is: urn:lsid:zoobank.org:pub:828C6915-3AA2-45AE-99C0-E97E7F6E1A0A. The electronic edition of this work was published in a journal with an ISSN, and has been archived and is available from the following digital repositories: PubMed Central, and LOCKSS.

## Results

### 1. Systematic paleontology

CETACEA, Brisson [47]

ODONTOCETI, Flower [48], *sensu* Fordyce and Muizon [26]

PAN-PHYSETEROIDEA, Velez-Juarbe et al. [9]

PHYSETEROIDEA, Gray [49], *sensu* Velez-Juarbe et al. [9]

*Albicetus oxymycterus*, new combination, urn:lsid:zoobank.org:act:0A979799-2E74-4C2E-9E23-8940A036AE18,

**Type and only known species:** *Albicetus*, nov. gen., *oxymycterus*

**Etymology:** Combining the Latin words *albus* (white) and *cetus* (whale). The name pays tribute to H. Melville [50]’s classic American novel *Moby-Dick; or*, *The Whale*. In the novel, Melville refers to Moby-Dick as “the White Whale”, a creature of “unwonted magnitude” with a “remarkable hue” and “deformed lower jaw” [50]. These traits are coincidentally similar to the type specimen of *Albicetus*, a white fossil sperm whale whose jaws have been displaced due to diagenetic processes, providing apt inspiration for the connection to the famous literary whale.

**Age:** Same as that of the only known species.

**Diagnosis:** Same as that of the only known species.

**Fig 5:**
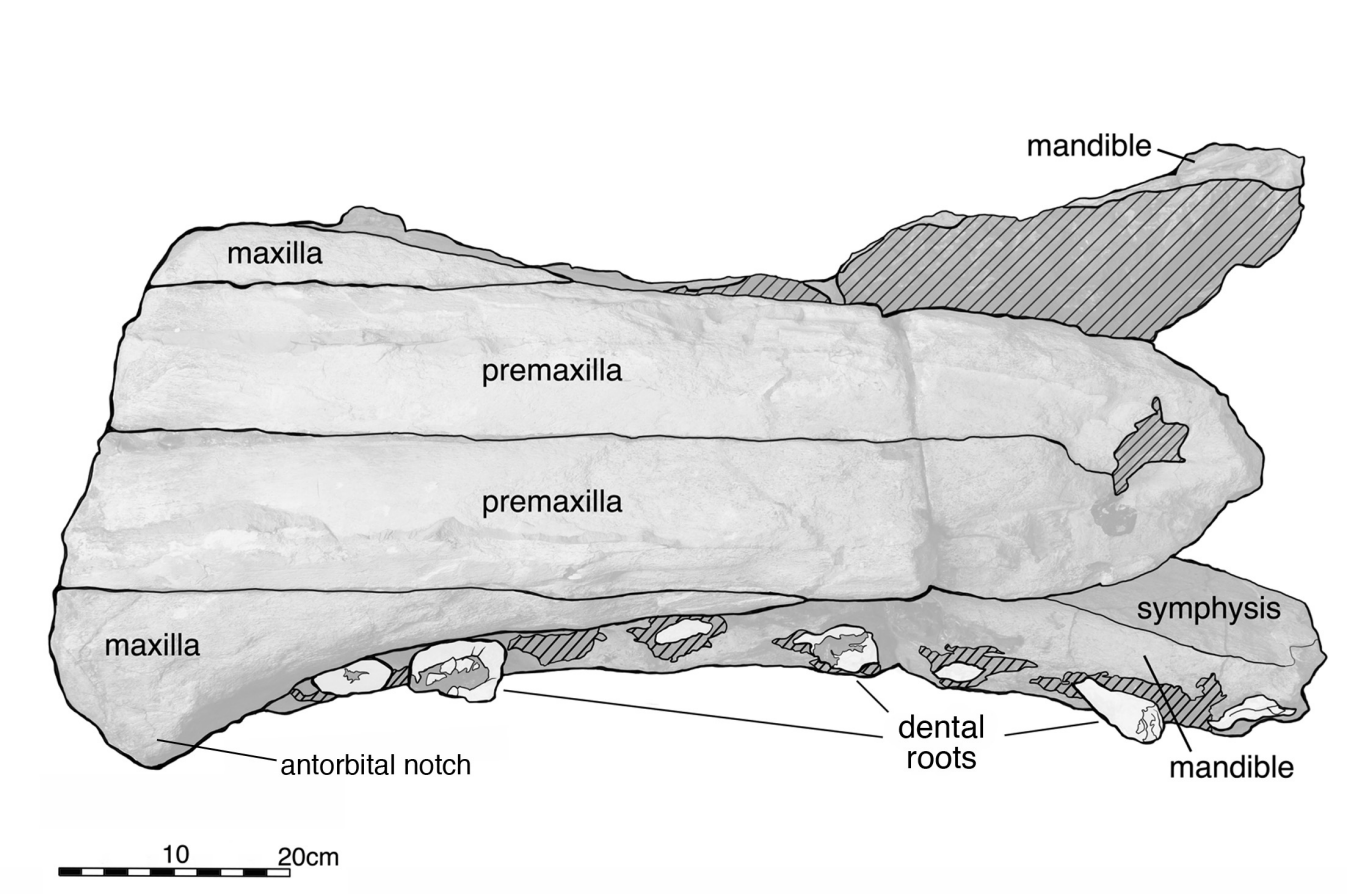
Dorsal view of the rostrum of *Albicetus oxymycterus* (USNM 10923). Illustrated with a low opacity mask and interpretive line art. Tooth fragments retained in the alveoli are emphasized with a nearly opaque white layer. To view the 3D model of the specimen, visit the Smithsonian X 3D website at (http://3d.si.edu).

**Fig 6:**
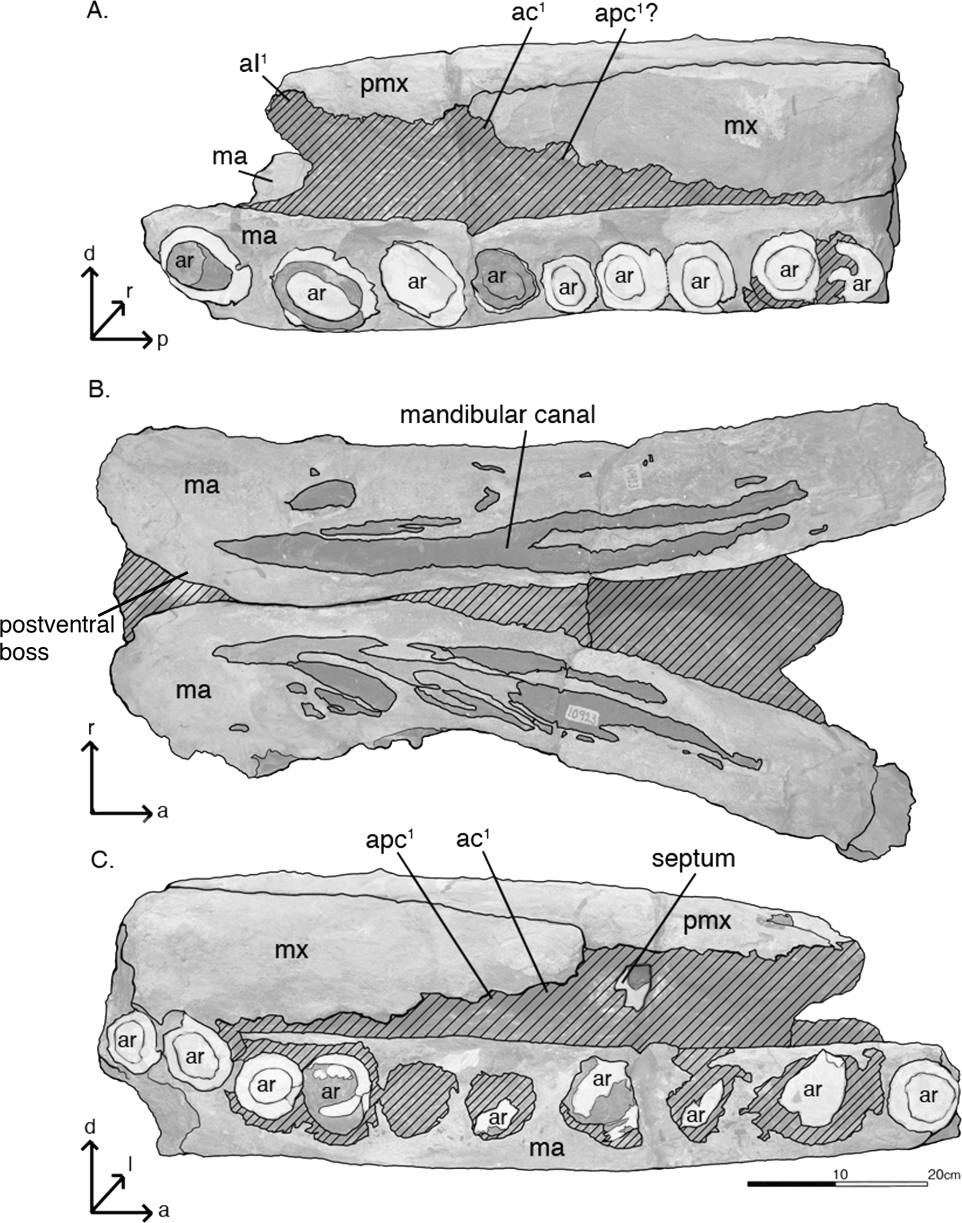
Lateral and ventral views of the rostrum of *Albicetus oxymycterus* (USNM 10923). Illustrated with a low opacity mask and interpretive line art. From top: A, left lateral, B, ventral, and C, right lateral views Abbreviations: *d* indicates dorsal direction, *l* indicates left lateral, *a* indicates anterior, *p* indicates posterior, *r* indicates right lateral. aI^1^ indicates first incisor alveoli, ac^1^ indicates first canine alveoli, apc^1^ indicates first post-canine alveoli, dr indicates alveolus with dental root. The mandibles (ma), maxillae (mx) and premaxillae (pmx) are all labeled accordingly. Tooth fragments in alveoli are emphasized with a nearly opaque white layer. To view the 3D model of the specimen, visit the Smithsonian X 3D website at (http://3d.si.edu).

**Fig 7:**
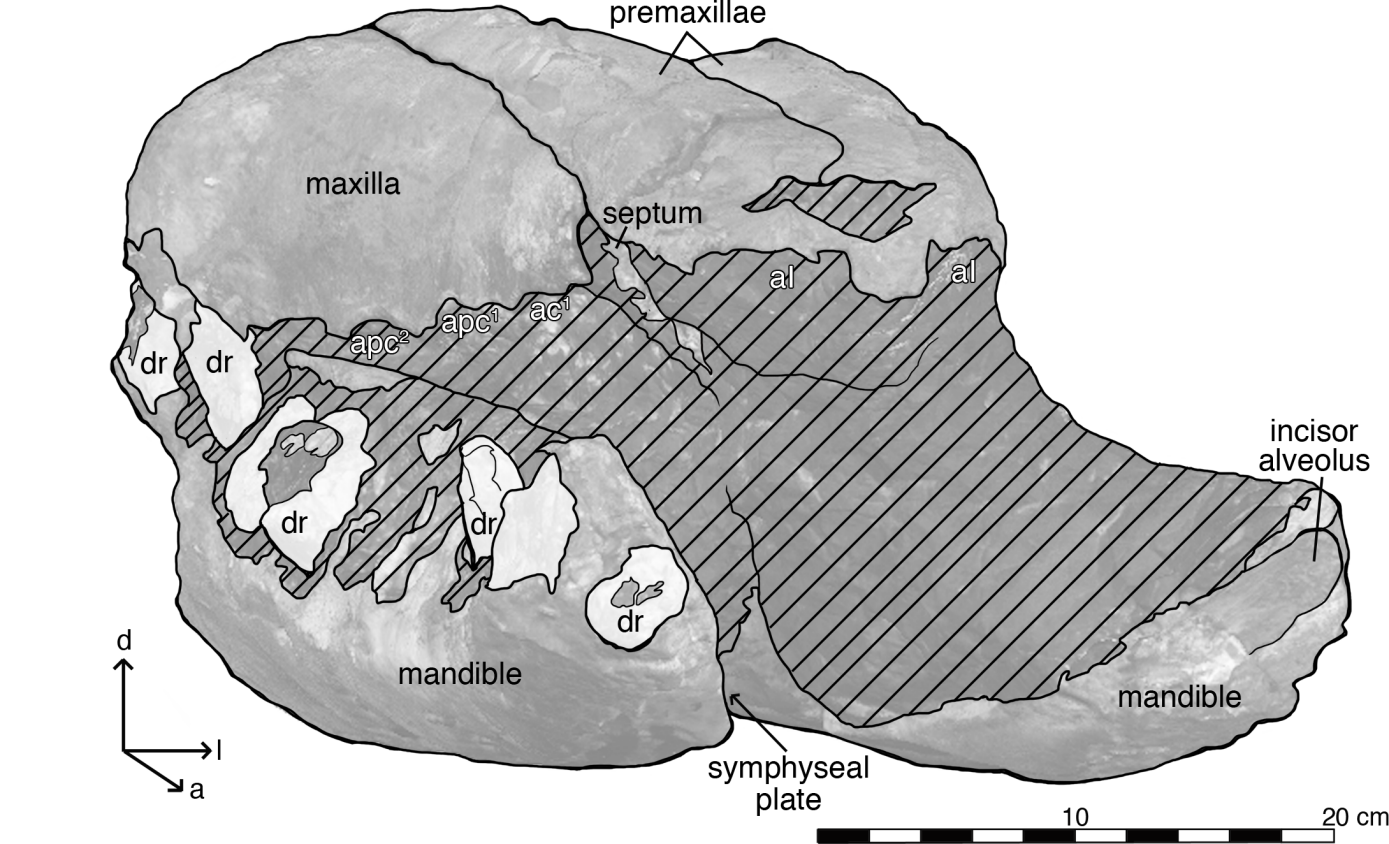
Oblique anterior view of the rostrum and mandibles of *Albicetus oxymycterus* (USNM 10923). Illustrated with a low opacity mask and interpretive line art. Tooth fragments retained in alveoli are emphasized with a nearly opaque white layer. Abbreviations: *d* indicates dorsal direction, *l* indicates left lateral, *a* indicates anterior, aI^,^, indicates incisor alveolus, ac^1^ indicates first canine alveoli, apc^1^ indicates first post-canine alveoli, dr indicates alveoli with dental roots. To view the 3D model of the specimen, visit the Smithsonian X 3D website at (http://3d.si.edu).

**Fig 8:**
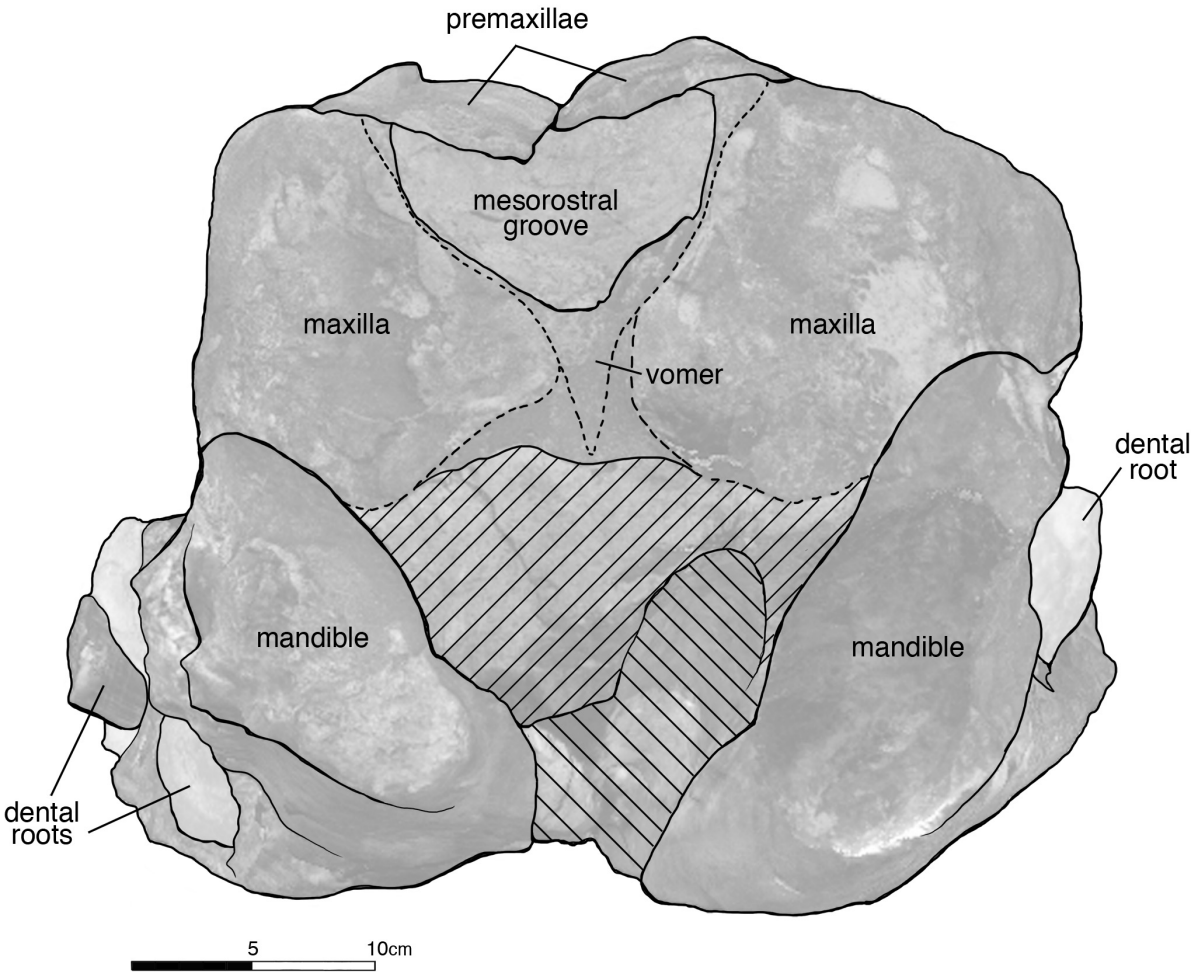
Posterior view of the rostrum and mandibles of *Albicetus oxymycterus* (USNM 10923). Illustrated with a low opacity mask and interpretive line art. Tooth fragments retained in alveoli are emphasized with a nearly opaque white layer. To view the 3D model of the specimen, visit the Smithsonian X 3D website at (http://3d.si.edu).

**Fig 9:**
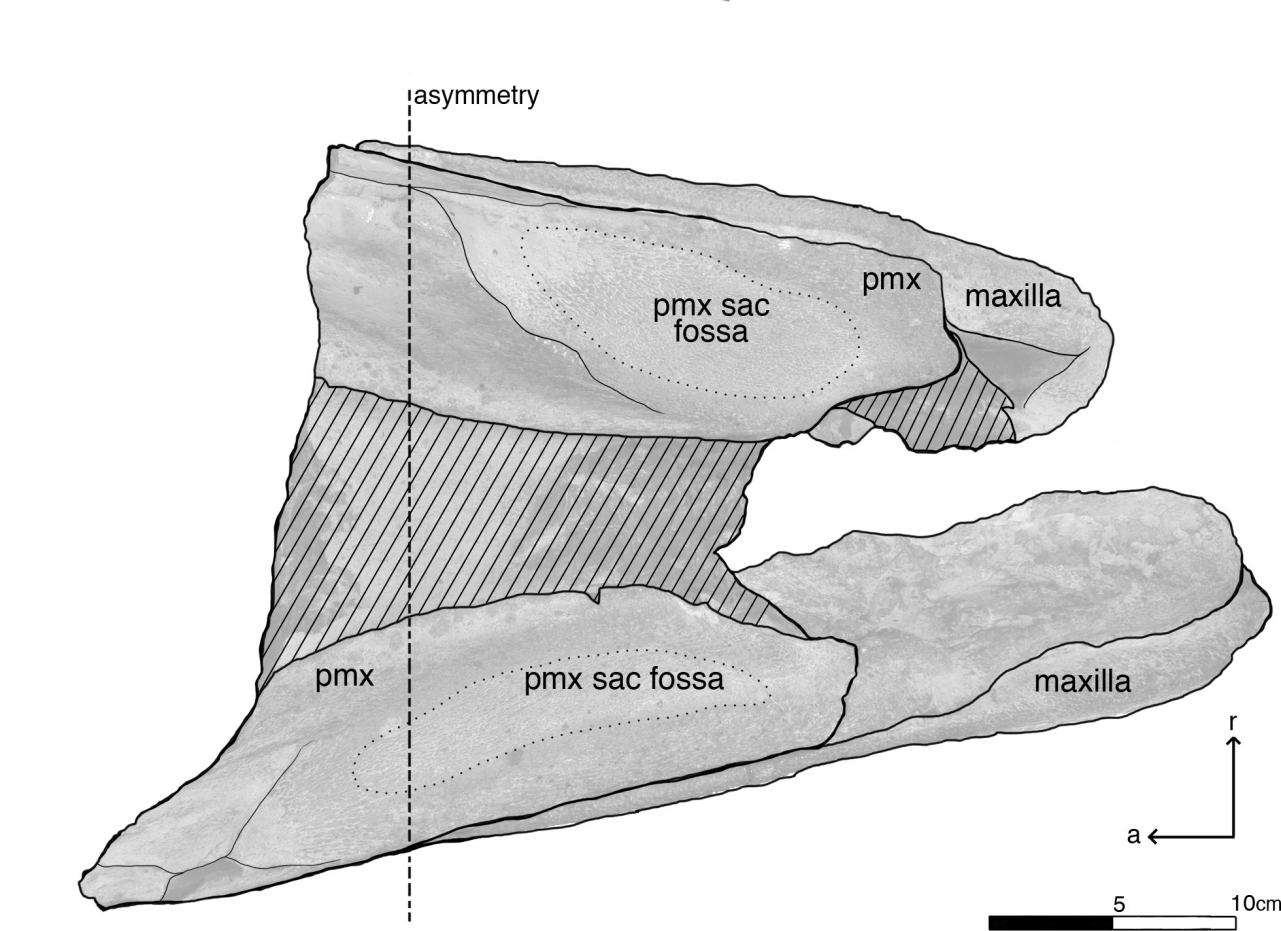
Isolated rostral fragment of *Albicetus oxymycterus* (USNM 10923) in dorsal view. Illustrated with a low opacity mask and interpretive line art. *a* indicates anterior direction, *r* indicates right lateral direction. The line of dashed line emphasizes the asymmetry in the premaxillae. The dotted line delineates the premaxillary (pmx) sac fossae, which are also asymmetrical. To view the 3D model of the specimen, visit the Smithsonian X 3D website at (http://3d.si.edu).

**Fig 10:**
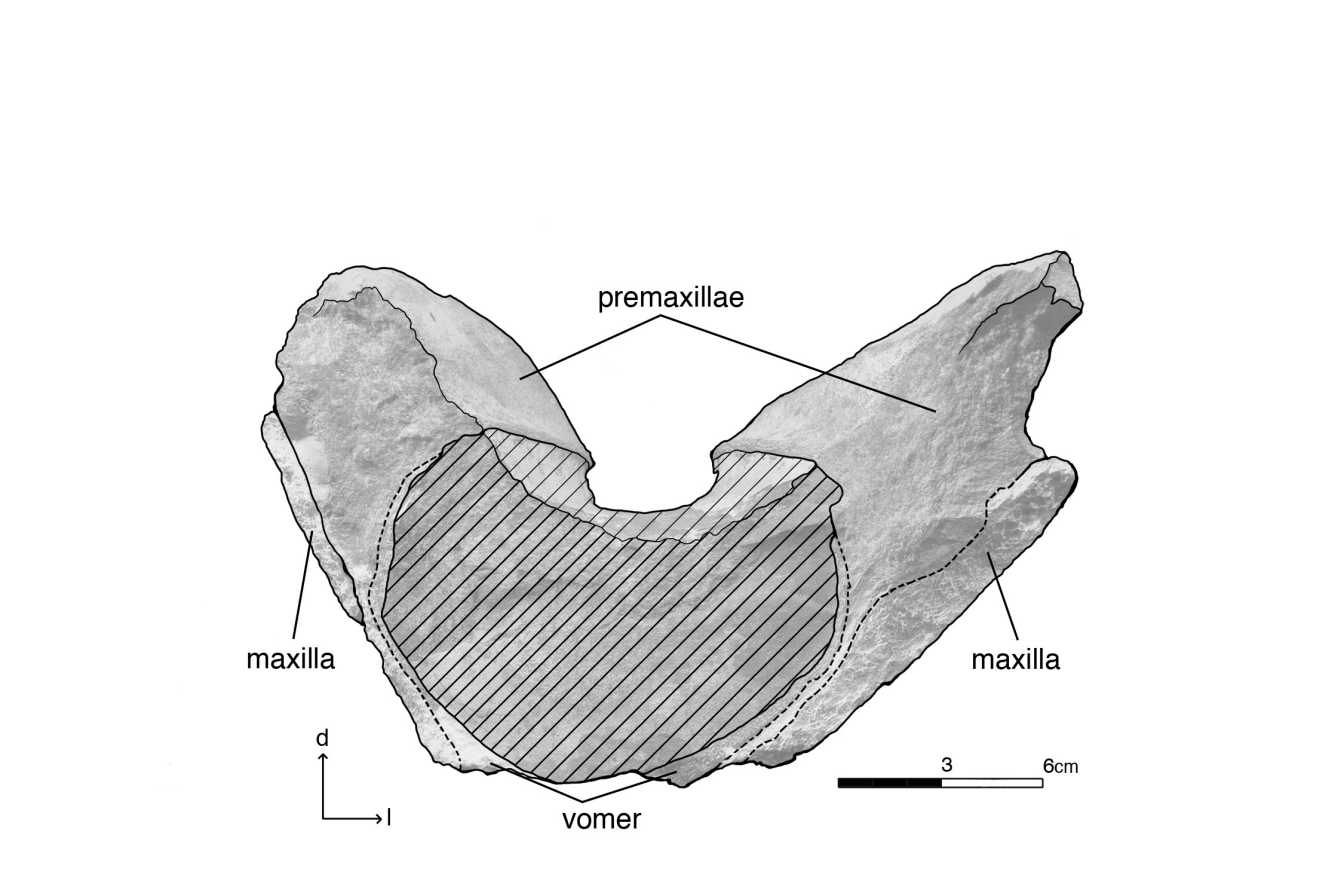
Isolated rostral fragment of *Albicetus oxymycterus* (USNM 10923) in anterior view. Illustrated with a low opacity mask and interpretive line art. To view the 3D model of the specimen, visit the Smithsonian X 3D website at (http://3d.si.edu).

*Albicetus oxymycterus*, new combination (Figs 2 and 5–10, Tables 1 and 2)

**Table 1:** Measurements for type specimen *Albicetus oxymycterus* (USNM 10923). Measurements for main rostrum segment, right mandible, and upper rostrum fragment of *Albicetus oxymycterus* (USNM 10923), in centimeters.

MEASUREMENTS	(cm)
**Rostrum**	
a) Midline length	81.9
b) Length from tip of premaxilla to premaxilla-maxilla suture	27.4
c) Width at antorbital notch	49.4
d) Width of premaxillae (right side x2) anterior to antorbital notch	21.8
e) Width of maxillae (right side x2) anterior to antorbital notch	14
f) Width of premaxillae at premaxilla-maxilla suture	20.7
g) Width of premaxillae at anterior end	17.2
**Right Mandible**	
a) Total length	91.6
b) Length from tip to beginning of symphysis	57.8
c) Length of symphysis	18.4
d) Depth at posterior end of symphysis	27.2
e) Width at anterior tip of mandible	13.1
f) Width at beginning of symphysis	17.2
g) Width at posterior end of mandible	15.5
**Separated Posterior Rostrum Fragment**	
a) Midline length	40
b) Anterior total width	24.5
c) Posterior total width	17.5
d) Anterior mesorostral groove width	9
e) Posterior mesorostral groove width	6
f) Anterior width of combined premaxilla and maxilla(left side)	7
g) Posterior width of combined premaxilla and maxilla (left side)	5

**Table 2:** Alveolar Measurements for type specimen *Albicetus oxymycterus* (USNM 10923). Measurements for alveoli in right mandible of *Albicetus oxymycterus* (USNM 10923), in millimeters.

Alveolus number	Anteroposterior diameter (mm)	Lingual-lateral diameter (mm)	Interalveolar space between alveoli and adjacent alveoli (mm)
**1**	50	54	12
**2**	73	70	26
**3**	100	122	37
**4**	93	62	26
**5**	102	90	16
**6**	80	85	5
**7**	98	84	0
**8**	120	80	0
**9**	108	80	0
**10**	98	75	0
**11**	72	58	N/A

**Holotype:** USNM 10923, the incomplete extremity of the rostrum and mandibles, with 10 or 11 roots or portions of teeth *in situ* in each mandible, several incomplete teeth fragments found loose in the matrix, and a separate piece of the upper rostrum. Archival typewritten notes at USNM state that the specimen was first observed by Charles O. Roe (1867–1923) when he was a boy, and was collected by him some thirty years later in 1909, implying an initial discovery as early as 1879. These notes are consistent with Kellogg [28]’s report about the type specimen’s discovery around 1884, and it being subsequently moved to Roe’s home in 1909. The specimen was received by USNM on 16 February 1924 from Roe’s wife, after his death in 1923 [51].

**Etymology:** According to Kellogg [28], *oxymycterus* derives from the Greek words *oxy* (sharp) and *mycter* (nose).

**Type locality:** The type specimen was collected approximately 3.5 meters above the high tide line of a sea cliff approximately 20 m in height, north or near the original Santa Barbara Lighthouse, Santa Barbara, Santa Barbara County, California, U.S.A. (N 34° 20'12", W 119° 43'20" according to Kellogg) [28]. Archival typewritten notes at USNM indicate that the “exact locality is between the [original Santa Barbara] lighthouse and Hope Ranch,” and that “[other] parts of the skeleton are still in the bank.” Hope Ranch today is a residential community of approximately 1,600 acres, but in the late 19^th^ century it was a large private property until the Southern Pacific Railroad purchased it for development in January 1876. Actual development on the terrain did not begin until 1923 [52]. It was likely during this period of time between purchase and development that C. A. Roe collected the type specimen (1879–1909), since the property lines of Hope Ranch abutted the original Santa Barbara Lighthouse.

The original Santa Barbara Lighthouse, however, was destroyed in an earthquake on 29 June 1929 [53], and Kellogg [28]’s published coordinates correspond to a location about 5 km directly south of the original lighthouse, offshore in Santa Barbara Bay. The published locality account by Kellogg [28] corresponds today to sea cliffs located near the property of the current lighthouse [54], which is 100 m northeast of the original lighthouse, at the following coordinates (N 34° 23' 44", W 119° 43' 23"). We argue that this general vicinity, within less than a 100 m radius, likely represents the type locality of *Albicetus oxymycterus* (Fig 1).

**Formation:** Kellogg [28] described the stratigraphic provenance of the type specimen of *Albicetus* as a unit of bituminous dolomite in the sea cliffs of Santa Barbara County, likely belonging the Monterey Formation. This description is consistent with Minor et al.’s [55] geologic mapping of the Santa Barbara Coastal Plain, which at the reported locality shows the underlying lowest three subunits of the marine siliceous and calcareous mudstone and shale belonging to the Monterey Formation. These marine rocks are mapped as the lower calcareous unit of the Monterey Formation [55]. Overlying marine terrace deposits of Pleistocene age in this area do not match the lithology of the matrix with USNM 10923.

**Age:** Minor et al. [55] summarized biostratigraphic findings for lower calcareous unit outcrops of the Monterey Formation in the Santa Barbara Coastal Plain. Benthic foraminiferal assemblages point to Relizian to Saucesian stages, and calcareous nannofossil zones CN1-CN5, with ages generally tending to be younger in the northwestern localities, and older heading to the southeast. For Santa Barbara Point, a locality less than 1 km due east of original Santa Barbara Lighthouse, Minor et al. [55] reported benthic foraminiferal assemblages consistent with Relizian and Luisian Stages of Kleinpell [56–57] and calcareous nannofossils of lower middle Miocene zone CN4. These data constrain the age of the type specimen of *Albicetus* to the early middle Miocene (~16–14 Ma), or Langhian.

**Diagnosis:** *Albicetus* is a large odontocete (about 6 m in total length) that belongs in Physeteroidea based on the following features: large, single-rooted upper and lower teeth, with a ratio of condylobasal length to greatest tooth diameter greater than 0.03; anterior elongation of the premaxillae onto the rostrum; sinistral asymmetry of the posterior processes of the premaxillae; and a posterior section of the rostrum that is wide and relatively flat. *Albicetus* differs from all other known physeteroid genera in the following combination of character states: retention of enameled lower tooth apices with longitudinal striations; large lower and upper teeth with a ratio of condylobasal length to greatest tooth diameter of approximately 0.05; a mesorostral groove completely roofed over at the level of the antorbital notches; and the premaxillae angled downward into the midline, creating a trough down the middle of the rostrum.

### 2. Description

The type specimen (USNM 10923) consists of an incomplete skull that includes a partial rostrum and incomplete mandibles, with a number of isolated tooth fragments also found in the surrounding matrix (Figs 2 and 5–10). Much of the area between the ventral surface of the rostrum and the mandibles is filled with very dense, heavy, and nearly aphanitic grey sediments. These sediments severely hinder any ability to further prepare matrix from bone, which is poorly differentiated from the surrounding matrix. The teeth are also especially soft and chalky, which hinders any further mechanical preparation. The specimen itself is extremely heavy, and the bones of the type specimen are heavily mineralized. The cortical surfaces are also extremely weathered and eroded, likely mostly from exposure as the specimen protruded from the cliff face, which lasted at least a couple decades. These wear patterns are too extreme to differentiate primary weathering from abrasion via transport, or other diagenetic effects [58]. Almost all the bone has been permineralized or replaced by phosphatization in deep-sea sediments, and, in some cases, the original shape of the bone is difficult to distinguish from the surrounding matrix, especially along in posterior view of the rostrum, where it contacts the mandibles.

**Fig 11:**
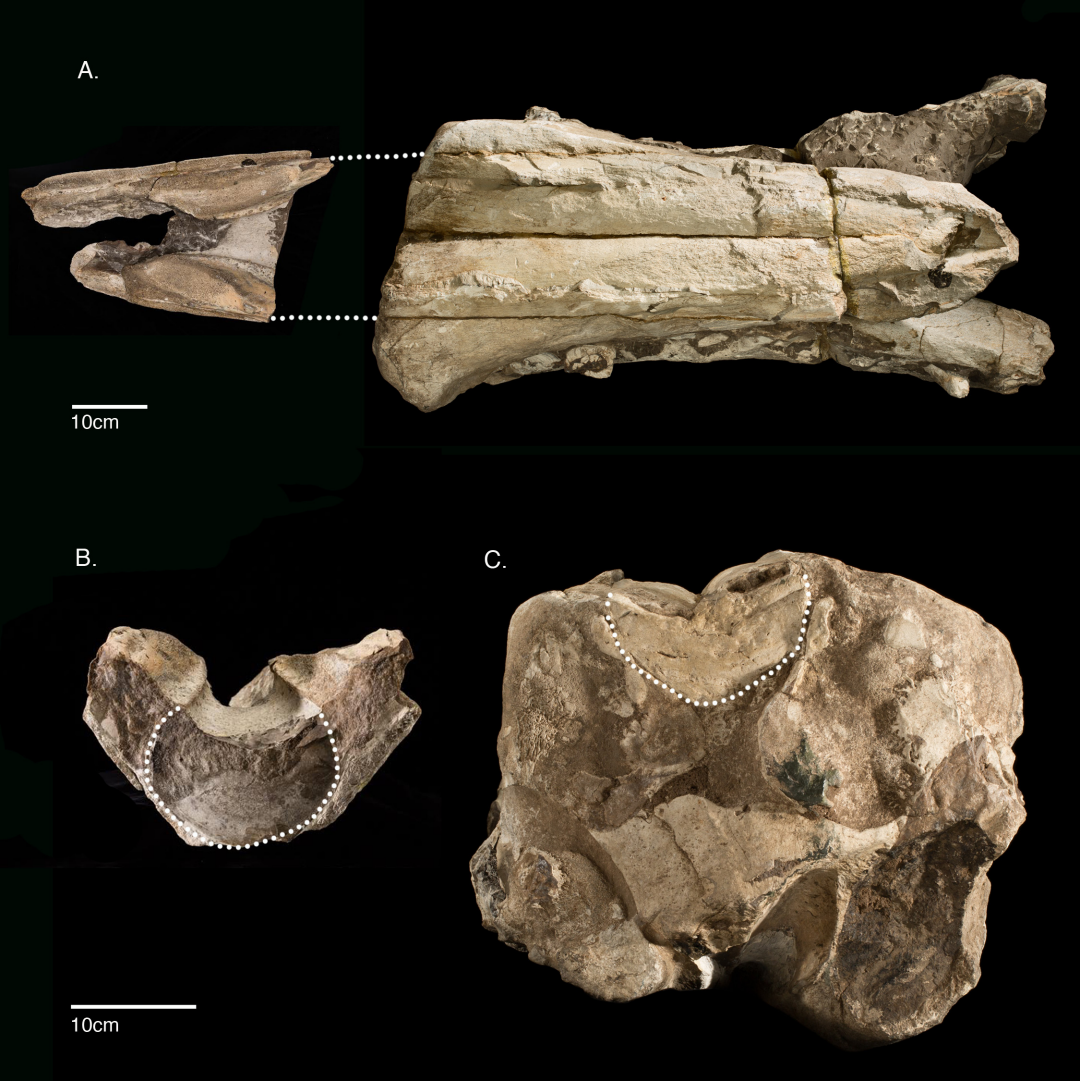
Suggested anatomical relationship between the isolated rostral fragment and the rostrum of *Albicetus oxymycterus* (USNM 10923). A, dorsal view of main rostrum section and upper rostral fragment. Dotted line indicated hypothesized non-direct connection between the main section and fragment. B, anterior view of upper rostral fragment and C, posterior view of main rostrum section. Both have a dotted line indicating the similarity between the transverse profiles of the vomer and mesorostral groove.

The left mandible shows the remains of 10 dental roots from teeth that have been fragmented through diagenesis, while the right has 11 (Fig 6A and 6C). The type specimen also includes an isolated fragment of the upper rostrum, which was not described by Kellogg [28] (Figs 9–11). This triangular fragment is composed of premaxillae, maxillae, and vomer, although it does not have direct, patent contact with the large rostrum.

Though sutures are indistinct, they appear to be closed. This, in combination with the size of the teeth and retention of dental roots in the mandibles, suggest that the specimen belonged to a mature whale.

#### Rostrum

Most of the specimen is composed of the rostrum, measuring 81.9 cm along the midline from the anterior termination of the preserved rostrum to a level slightly anterior of the antorbital notches (Fig 5). The slight tapering, the extent of the preserved mandibles displaced anterior relative to the rostrum (Fig 6), and the presence of an alveolar root on the anterior extremity of the right mandible (Fig 7), all suggest that the anterior termination of the rostrum is real. The rostrum is laterally compressed anteriorly, but it gradually widens towards the posterior along the maxilla, until it suddenly flares out laterally at the level anterior to the antorbital notches (based on similar dorsal profiles in other fossil physeteroids such as *Aulophyseter* and *Orycterocetus*) (Table 1). In posterior view, the dorsal surface of the maxillae curve ventrally from their line of contact with the premaxillae to the lateral margins, suggesting that the supracranial basin is not anteriorly elongated (Fig 8). Dorsally, the rostrum is composed primarily of the premaxillae and maxillae, and transverse sections of the vomer are visible in posterior view. All of the surfaces of premaxillae and maxillae are heavily eroded, with the cortical bone completely worn away. The right side of the rostrum is better preserved than the left as it reaches further posterior, therefore most of the descriptions of the premaxillae and maxillae are based on this side. Both right and left premaxillae and maxillae show some equivocal evidence for upper alveoli (see Dentition, below, for more remarks).

In dorsal view, the premaxillae dominate the majority of the rostrum, alone forming the anterior ~30% of the rostrum (Fig 5). The premaxilla-maxilla suture is about 27.4 cm posterior from the anterior tip of the rostrum, where the premaxillae measure 20.7 cm wide. Anterior to the premaxillae-maxillae suture, the premaxillae narrow in width to the tip of the rostrum, and maintain approximately the same width from the suture to the posterior end of the specimen. Although sutures are indistinct, the right and left premaxillae meet at the midline for the entirety of the preserved rostrum, roofing over the mesorostral groove (visible in posterior, transverse section (Fig 8). No premaxillary foramen is visible on either premaxilla. In lateral view, the premaxillae gradually thin dorsoventrally from the anterior extremity to the posterior end of the rostrum (Fig 6A and 6C). In posterior view, the premaxillae shows a slightly arched profile, angling downwards towards the midline to create a trough down the middle of the rostrum (Fig 8). On this portion of the rostrum, there is no patent asymmetry in the dimensions of the right and left premaxillae (see description of isolated rostral fragment for further discussion of asymmetry).

In dorsal view, the maxillae are most narrow where they meet the premaxillae, 27.4 cm from the anterior tip (Fig 5). From this level, the maxillae gradually widen until the posterior of the rostrum, where they rapidly expand laterally to a width of at least 14 cm from its medial margin (as measured from the right maxilla) along the maxillary flange (sensu Mead and Fordyce [42: 62]). Based on comparison to other fossil physeteroids, this is just anterior to the antorbital notch. In lateral view, the maxillae increase in dorsoventral depth from the premaxilla-maxilla suture towards the base of the rostrum (Fig 6A and 6C). In posterior view, the maxillae underlie the premaxillae, meeting the latter dorsally, though this relationship may be due to increased erosion further from the midline (Fig 8). From the lateral margin in posterior view, the maxillae curve dorsally towards the midline to meet the premaxillae.

The mesorostral groove is not visible in dorsal view, being completely sealed over by the premaxillae (Fig 5). In posterior view, the vomer forms the floor of this basin, along with the lateral walls of the mesorostral groove (Fig 8). Though it is unclear whether the mesorostral groove was ossified; in posterior view, it is now filled with sediment, and was dorsoventrally low in life, as in living *Physeter*. See description of the isolated rostral fragment for more on the vomer and mesorostral groove further posterior to the antorbital notch.

#### Mandibles

The mandibles are both incomplete, but they preserve the entire mandibular symphysis, along with mandibular rami 15 cm posterior of the symphysis. Although the extremities of both mandibles end abruptly, the anterior orientation of the anterior-most alveoli (likely incisors) suggests that the type specimen preserves most of the anterior portion of the mandibles in life (Fig 7). The orientations of the right and left mandibles are not preserved in life position, especially relative to the anatomical planes of the overlying rostrum (Fig 5). From 57.8 cm posterior of their anterior tips, the right and left mandibles are disarticulated, exposing the symphyseal plate along the medial surfaces of the right mandible, whereas the counterpart surface on the left mandible remains covered in sediment (Fig 7). Both mandibles are also displaced anteriorly relative to the overlying rostrum, so that they extend past the tip of the rostrum (Fig 5). In dorsal view, the mandibles have also been rotated outwards, so that the alveoli face dorsolaterally and the medial surfaces of the mandibles face the ventral surface of the rostrum. The dorsal curvature of the preserved anterior margins of the mandibles, along with the anterior orientation of terminal alveoli, suggest that, in life, the mandibles had a lateral profile that gradually curved dorsally along the symphysis to the anterior tip, however there is the possibility that this morphology is diagenetic from sediment compaction and deformation (Fig 6A and 6C). There is no evidence of the presence of mental foramina on either mandible.

The right mandible retains evidence of 11 alveoli, while the left retains evidence of 10 (Fig 6). Both mandibles are heavily eroded on the ventral side. The rami of the mandibles, posterior of the symphysis, remain laterally flattened at the level of the base of the rostrum. Although the bone is very difficult to separate from matrix at visual inspection, we suspect that the dorsal rise of the mandibles at this level (Fig 2E, Fig 6B) possibly represents the beginning of the acoustic pan bone region. Given all of the aforementioned observations, we propose that the majority of the mandibles are indeed preserved with the rostrum.

In ventral view, numerous black, branching, and likely phosphatized infilled canals about 2–3 cm wide, extend along the ventral surface of the mandibles (Fig 6B). Ostensibly, these features are exposed from erosion of the overlying cortical bone, and likely represent branches of the mandibular canal. In life, these branches would have once housed nerves and the inferior alveolar arteries connecting to alveoli of the lower dentition, although to date there are only published descriptions of cranial and postcranial sperm whale arterial systems to evaluate this notion, with no particular detail on physeteroid mandibles [59]. Towards the posterior end of the mandibles, there is a patent posteroventral boss on the right mandible, implying the termination of the symphysis in life.

#### Isolated rostral fragment

Included with the type specimen is a large cranial fragment that was collected in the surrounding matrix, representing a posterior portion of the rostrum separate from the skull and mandible block (Figs 9 and 10). This fragment measures 39.5 cm down its anteroposterior length, narrowing from 25 cm wide at its anterior end to 16.5 cm at its posterior termination (Fig 9). Although it does not have a patent contact with the larger portion of the rostrum, the plane of the anterior breakage is subtransverse, and we suspect it would have articulated within 5–25 cm of a gap with the larger portion (Fig 11). The fragment consists of the premaxillae along the dorsal surface, showing putative premaxillary sac fossae, overlying sediment infilling the mesorostral groove, and finally, along the ventral side, a thin layer of vomer and maxillae along the lateral aspect. The narrowing of the premaxillae and maxillae posteriorly suggests that both are incomplete laterally.

In dorsal view, there are no premaxillary foramina visible (Fig 9). The right and left premaxillae of the fragment are asymmetrical, with the left premaxillary sac fossa showing a more elongate outline in dorsal view (around 17 cm lengthwise) and being displaced anteriorly by about 10 cm relative to the right one (around 14 cm lengthwise) (Fig 9). This asymmetry is consistent with the sinistral displacement of skulls bones near the narial opening in physeteroids [15,16, 22]. The dorsal surfaces of the premaxillae are slightly concave and eroded, but towards the posterior end more cortical bone is visible grading through to the underlying maxillae. Ventrally, the vomer bridges the right and left premaxillae and maxillae via the mesorostral groove, which is mostly filled with a thick layer of matrix (Fig 10). At this level posterior, the mesorostral groove is open, separating the premaxillae by 9 cm at the anterior end and 6 cm and the posterior end. We can therefore say that the mesorostral groove was roofed over by the premaxillae from the antorbital notches anterior to the tip of the rostrum as seen in the main rostrum fragment (Fig 5), but that the mesorostral groove opens within 5–25 cm posterior of the antorbital notches (Fig 11).

#### Dentition

**Fig 12:**
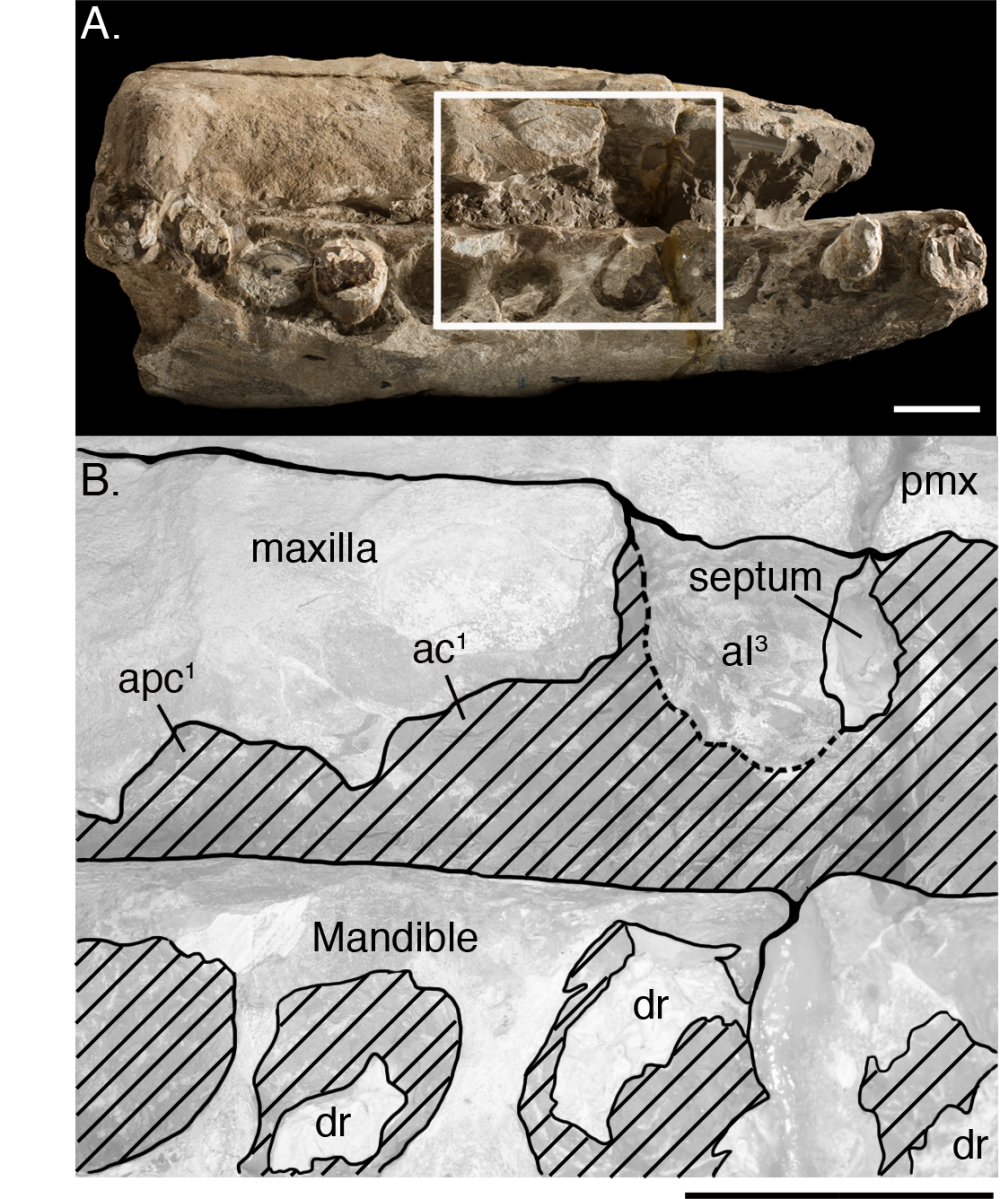
Upper alveoli. Right lateral close up of septal bone material retained in the third premaxillary incisor alveoli (A) of *Albicetus oxymycterus* (USNM 10923), and a illustration of the same area with a low opacity mask and interpretive line art (B). Scale bar measures 10 cm in both A and B.

Kellogg [28,60] argued that *Albicetus* very clearly possessed upper dentition, and we agree with these observations, although they require a careful reading of the available morphological evidence of the type specimen to dissuade immediate skepticism. First, Kellogg [28] noted that premaxillae both housed three upper alveoli, a eutherian trait inherited by all odontocetes. The clearest evidence for anterior-most alveoli, corresponding to I1, can be observed on the left premaxilla, which shows a deep, broad arc along its anterolateral margin infilled with sediment (approximately 8 cm in diameter) (Fig 2D). There is no evidence for a tooth or tooth root at this position. The right premaxilla is heavily eroded, but most clearly shows an alveolus where I3 would be located, which was already deeply excavated by manual preparation in 1924. This extends nearly 10 cm in diameter, just anterior to the level of the premaxilla-maxilla suture on this side (Fig 12). Both the alveoli at I1 on the left premaxilla and I3 on the right premaxilla would have been oriented ventrally, though I1 may also have been tilted slightly anterior. A small plate of bone, ostensibly belonging to an alveolar septum between the right I2 and I3, is visible in a lateral profile on this side (Fig 12). The inference of any other upper alveoli in the premaxillae is otherwise difficult to ascertain.

Kellogg [28,60] also indicated that 8 alveoli were present in the “distal end” of each maxilla, and he argued that as many as 18 upper teeth would have been present in life. The evidence for upper alveoli in the maxillae is difficult to pinpoint in lateral view, as Kellogg [28] noted, given the diagenetic wear to the specimen. However, a regular scalloped pattern suggesting alveoli can be observed along the lateral margin of the maxillae, with a more prominent pattern on the right side than the left (Fig 12). Despite the poor condition of the bony surface of the rostrum, the visible morphology of the lateral margin of the maxillae is largely consistent with much better preserved fossil physeteroids, such as *Acrophyseter deinodon* Lambert et al. [61], which shows a similar pattern with *in situ* upper dentition [21]. At close inspection, clear septa separate the anterior-most alveoli of the right maxilla, especially for C1 (first canine) and PC1 and PC2 (post-canines 1 and 2) (Fig 12). At most, we count 6 alveoli for the upper right maxilla, and no more than 5 alveoli for the upper left maxilla, all oriented ventrally and with diastema of at least 1 cm between each (Fig 6A and 6C).

Assuming that 3 alveoli were present in each premaxilla, the maximum upper tooth count was 9 teeth, per quadrant, consistent with Kellogg’s [28] estimate of “18 or more teeth carried in each jaw.” There are, however, no intact upper teeth, nor intact preserved upper tooth roots visible in the rostrum of *Albicetus*. Medium to large size fossil physeteroids (e.g., *Acrophyseter*, *Livyatan* Lambert et al. [5]) are now known with conspicuous upper dentition and alveoli [5,21,61] that broadly matches their lower dentition in size and morphology. This may have been the case in *Albicetus*, as the size of the upper alveoli is on a similar scale as the lower alveoli (Fig 12). We also note that all of the intact and isolated teeth share similar size ranges with intact lower dentition. Kellogg [28] argued that the isolated teeth fragments found with the specimen belong to the upper dentition, having fallen out of the rostrum due to gravity during decay. We cannot confirm this argument, since the surviving fragments of isolated teeth found with the specimen neither connect with the matrix surrounding the rostrum, nor with any of the existing surfaces of the lower dentition. It is possible that *Albicetus* possessed slightly smaller upper dentition, in the trend of vestigial upper dentition in some fully mature *Physeter* males [62], but the rostrum of *Albicetus* is simply too large and dense for any currently available x-ray computed tomography technology to non-destructively penetrate and conclusively reveal upper dentition in the matrix ventral to the rostrum.

The lower alveoli in the mandibles of *Albicetus* are very large, occupying more than half the lateral width of the mandible. The transverse sections of the alveoli along the mandibular border are more ovate towards the anterior end of the mandibles, and more circular posteriorly, likely indicating that the anterior-most teeth were more obliquely oriented than the posterior teeth (Fig 6A and 6C). Most of the alveoli are filled with matrix, but several retain tooth roots. These alveoli have matrix filled in around the roots around 1–2 cm in thickness, suggesting that, in life, the gum tissue would have actively kept the teeth rooted in the alveoli. Each alveolus is distinct from one another, with spacing ranging from virtually none to 3.7 cm.

Several incomplete teeth, currently free of sediment, were reportedly collected in the matrix surrounding the rostrum specimen by Kellogg [28], and almost all are missing tooth crowns and apical tips. These teeth are large, measuring up to 20.5 cm in length (with the crown missing) and up to 8 cm in diameter. In cross-section, they are composed of an internal cone of ossified dentine surrounded by a thick layer of cementum.

**Fig 13:**
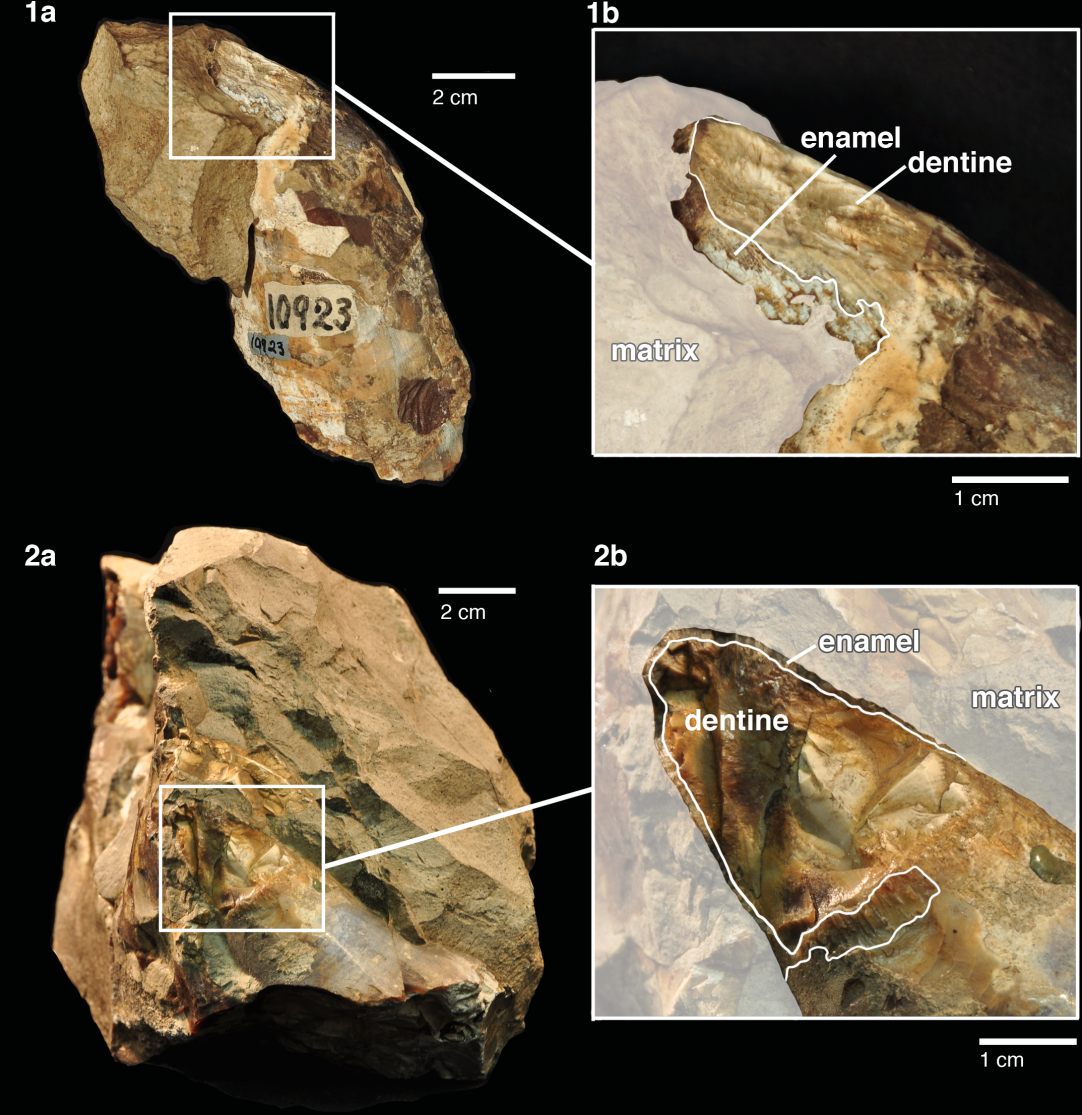
Isolated teeth found in the matrix, showing enamel caps of *Albicetus oxymycterus* (USNM 10923). Both show remnants of enamel tooth caps with coarse, longitudinal striations. Other isolated tooth fragments found with the specimen were too fragmentary to warrant illustration, with the exception of the tooth fragment shown in Fig 15. To see the 3D model of the enameled tooth (A), visit the Smithsonian X 3D website at (http://3d.si.edu).

Kellogg [28] noted the presence of a “third mandibular tooth…[that] broke away from the end of the root in the mandible at the time the specimen was removed from the sea cliff [and] measures 153 mm. in length.” This specimen was not illustrated in either of Kellogg publications [28, 50], and we have not been able to match any of the surviving isolated teeth with broken teeth in any of the anterior tooth positions of the mandible. Although the illustrated tooth in plate 9 of Kellogg [60] is suggestive of enamel apices for the teeth of *Albicetus*, the details of the enamel cap are poorly reproduced. Among the surviving material, we did observe two teeth still embedded in small bits of matrix that retain their tips, and show enameled crowns (Fig 13). The enamel clearly shows coarse, longitudinal striations (Fig 13). The crown has a length of 2.3 cm and the enamel is around 1 mm thick. No constriction of the neck is present below the crown, where the tooth measures 6.5 cm in diameter (Fig 13).

### 3. Body size estimates results

**Fig 14:**
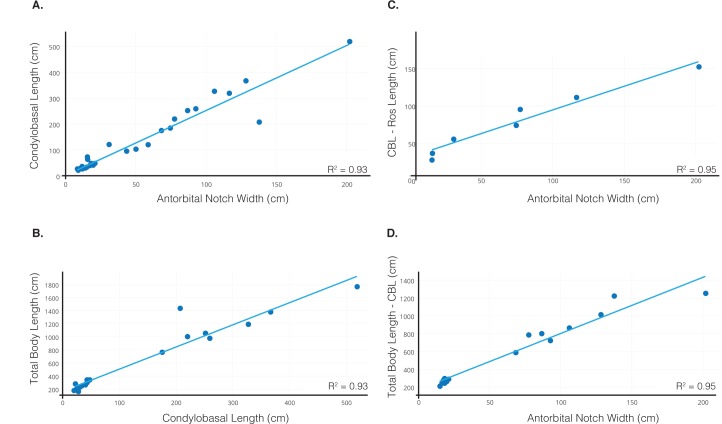
Allometric regressions of specimen-based datasets estimating condylobasal length and total length of *Albicetus*. A, condylobasal length from 36 fossil and extant physeteroids against antorbital notch width, to estimate a condylobasal length for *Albicetus*. B, condylobasal length against total body length, to estimate a total length for *Albicetus*. C and D follow the same methods as graphs A and B respectively, but take into account varying rostrum lengths.

The two methods of bivariate OLS regressions used to calculate condylobasal length and total length of USNM 10923 produced an estimated body size for each measurement, with the first method providing a lower bound estimate, and the second method providing an upper bound estimate. The first regression calculated condylobasal length (126.8 cm) using an antorbital notch width of 49.4 cm with the following equation (Fig 14A):

CBL=(2.51)×AON+2.84 Method 1, Eq 1

Our second regression for condylobasal length, taking into account variable rostrum length, produced the following equation (Fig 14C):

(CBL-RostrumLength)=(0.634)×AON+31.2 Method 2, Eq 1

With this equation, we used the preserved rostrum length of 81.9 cm to calculate a condylobasal length of 144.4 cm, yielding a presumed upper bound estimate for this measurement. We then used each CBL estimate to calculate a total length (TL) for *Albicetus*, using a specimen-based dataset grounded in corresponding skull and field measurements for living *Physeter* and *Kogia* spp. Our lower bound TL estimate was calculated as follows, using our first CBL estimate, yielding 592.2 cm (Fig 14B):

TL=(3.4)×CBL+161 Method 1, Eq 2

We generated an upper bound TL estimate by using antorbital notch width and our second CBL estimate, and similarly taking into consideration variable rostrum length, with the following equation (Fig 14D):

(TL-CBL)=(6.33)×AON+31.2 Method 2, Eq 2

**Fig 15:**
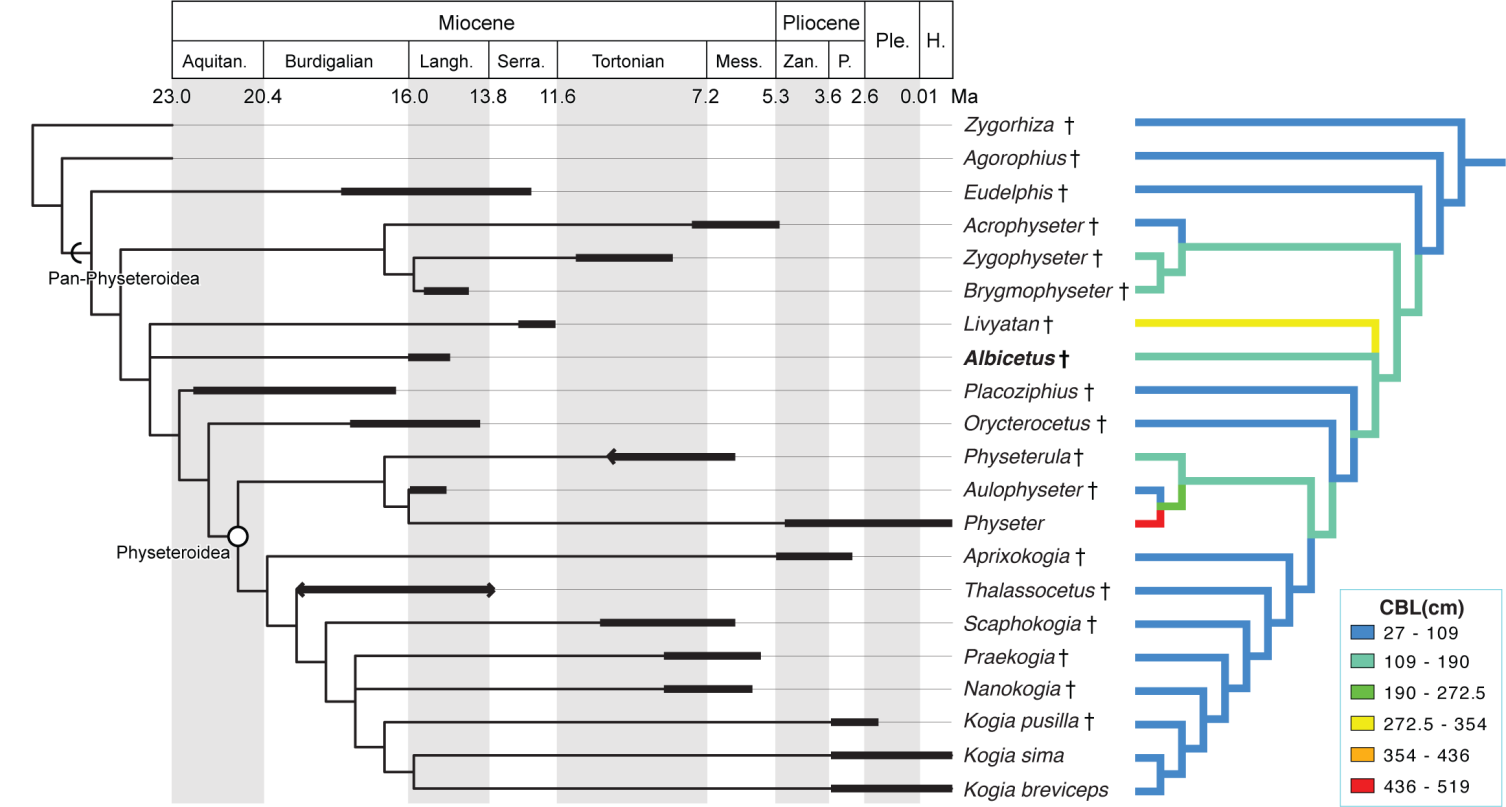
Phylogenetic results of Physeteroidea, calibrated for geologic time (left) and illustrating changes in body size (right). The left tree shows the phylogeny with black bars corresponding to the stratigraphic ranges of each taxon. Arrows on the end of black bars indicate lower confidence in stratigraphic boundaries. The right tree shows the evolution of body size in physeteroids, mapping condylobasal length as a proxy for total length, binned by 6 color values.

This latter equation yielded a total length of 627.1 cm. Thus, our estimated condylobasal length of USNM 10923 is between 126.8 cm and 144.4 cm, and our estimated total length is between 592.2 cm and 627.1 cm. We used condylobasal length estimates (proxies for total length) for all of the cetacean taxa (see S4 Table) as trait values and mapped them on the consensus tree, using squared change parsimony [63]. These total length trait values were categorized by color at regular intervals, providing the basis for visualizing the evolution of body size among different lineages of physeteroids, including *Albicetus* (Fig 15).

### 4. Phylogenetic analysis results

The phylogenetic analysis produced a strict consensus tree of 15 trees with a tree length of 100, consistency index of 0.585, and retention index of 0.715. In the consensus tree, *Albicetus* was placed among stem Physeteroidea, forming a polytomy with *Livyatan*, all crown-ward pan-physeteroids, and all crown physeteroids (Fig 15). The bootstrap values range from 85 for Physeteroidea without the outgroups of *Zyghorhiza* and *Agorophius* Cope [64], to 52 for the clade of *Zygophyseter*+*Brygmophyseter*. Kogiidae as a whole received a bootstrap value of 58, with *Kogia breviceps+Kogia sima* receiving a value of 77 (See S1 Fig for a phylogenetic tree with bootstrap values). Overall, these statistics suggest low support for the relationships recovered in our analysis, although our results are consistent with previous work (e.g., [5]).

## Discussion

### 1. Redescription of the type specimen of *Albicetus oxymycterus*

**Fig 16:**
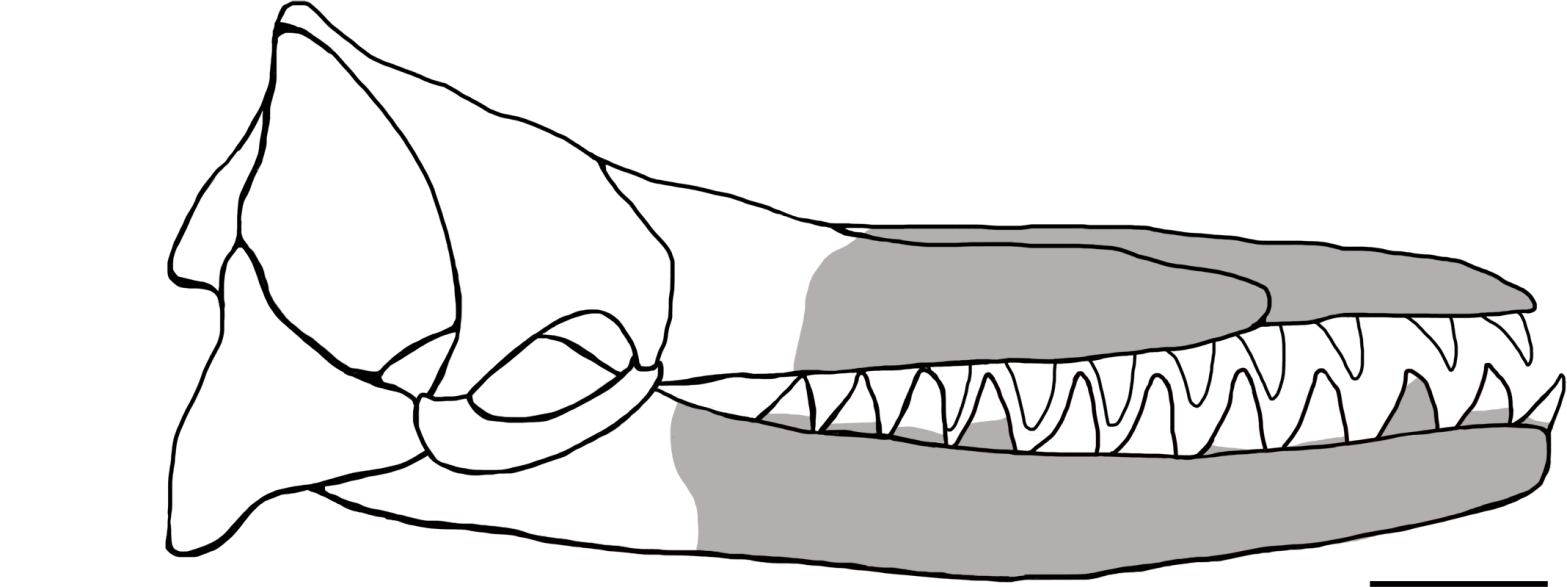
Skeletal reconstruction in right lateral view of the skull of *Albicetus*. Grey shaded area represents main rostrum, mandible, and tooth material present in the holotype specimen. The upper rostral fragment material is not shown as it would not be visible in lateral view. Scale bar measures 20 cm.

Our redescription of USNM 10923 differs from the original description by Kellogg [28] in the addition of a description of the separate posterior rostral fragment, body size estimates (Fig 14), and a phylogenetic analysis. We also expanded on the two photos of the specimen in Kellogg’s publications with a suite of descriptive Figs (Figs 5–13), including a 3D model (Fig 4), a skeletal reconstruction (Fig 16), and a phylogenetic tree mapping the evolution of body size among the Physeteroidea (Fig 15). Our description likely contrasts most strongly with Kellogg’s in the observations of the upper dentition. In *Livyatan*, *Acrophyseter*, and *Brygmophyster—*all stem physteroids—the large upper teeth in the proximal region are set in from the lateral margin of the maxillae by at least a couple centimeters [21, 61, 65]. In the case of *Acrophyseter*, there is even evidence of extra bony outgrowths along the alveoli in the maxillae acting as buttresses to support large upper teeth [61]. None of these features are preserved or present in *Albicetus*, with the ventrolateral margins of the maxillae in lateral view showing semi-circular indentations along the margin consistent with alveoli (Fig 12). We do not think that these indentations are the result of mechanical preparation (by C. A. Roe or perhaps USNM staff) in search of teeth or alveoli, although some of the alveoli (e.g., right C1) are certainly excavated of any infilled sediment, as opposed to others (e.g., left I1). Some of the preserved morphology may have formed during diagenesis, as the mandibles were forced apart and rotated laterally or outwards by compaction or gravity and the weight of the overlying rostrum pressed down on the mandibles. For example, in right lateral view, the posterior lower teeth directly abut the ventral surface of the maxilla, forming semi-circular indentations (see Figs 6C and 12), which appear to show little morphological distinction between bones belonging to the upper versus lower alveoli. As noted above, it is unclear whether the surviving isolated teeth collected in association with the type specimen belong to the upper or lower dentition, although Kellogg [28] maintained the former.

### 2. Morphological comparisons among physeteroids

The fossil physeteroid that most resembles *Albicetus* in general size, shape, and characteristics is *Aulophyseter morricei* (holotype USNM 11230), described by Kellogg [25] from the Sharktooth Hill bonebed of the middle Miocene Round Mountain Silt, in California. The largest known skulls of *A*. *morricei* are on a similar scale as *Albicetus*, with the midline length of the rostrum reaching 121.2 cm and the antorbital notch width estimated at 58.7 cm in BE 7791/1, the largest known specimen of this taxon. The premaxillae of *A*. *morricei* are similar to that of *Albicetus*, making up most of the rostrum and dominating the distal end. As with *Albicetus*, the premaxillae widen posterior from the tip to the premaxilla-maxilla suture, and then maintain a similar width further posterior in dorsal view (Fig 5). *A*. *morricei* has the mesorostral groove roofed over by the premaxillae for most of the length of the rostrum, as in *Albicetus*. However, in *Aulophyseter*, the mesorostral groove opens anterior to the antorbital notches. This situation differs from *Albicetus*, where the mesorostral groove opens posterior to the antorbital notches. In lateral view, the maxillae in *A*. *morricei* narrow from the posterior end to the premaxilla-maxilla suture where they are narrower than the premaxillae, similar to *Albicetus* (Fig 6A and 6C). From the premaxilla-maxilla suture in dorsal view, the maxillae flare out laterally towards the antorbital notches, but the widening directly anterior to the antorbital notches in *A*. *morricei* is not as pronounced as in *Albicetus* (Fig 5). The maxillae of *A*. *morricei*, which curve ventrally towards the midline to meet the premaxillae, also differ from the maxillae of *Albicetus*, which curve dorsally towards the midline. The concave shape of the maxillae in *A*. *morricei* forms the lateral wall of the anteriorly elongated supracranial basin, a feature that is not suggested in *Albicetus* due to the convexity of the maxillae (Fig 8).

Kimura et al. [4] pointed out that the teeth referred to *A*. *morricei* by Kellogg [26] in his original description were too large to fit into the alveolar grooves on the palate of the referred skulls. Kimura et al. [4] identified them as not sperm whale teeth, but as teeth belonging to desmatophocid pinnipeds, likely *Allodesmus* spp., which are abundantly represented in the same lithologic unit, including with cranial remains [39–40]. Kimura et al. [4] provided the first evidence for the real tooth morphology of *A*. *morricei* by illustrating a single isolated tooth associated with a diagnostic left mandible (LACM 42816), from the same lithologic horizon, which shares morphological similarities with the holotype. The associated tooth, which has a comparatively small diameter, lingual curvature, and lacks enamel, also fits directly into the small alveoli of this specimen. Therefore, despite cranial similarities, *Aulophyseter* and *Albicetus* possess starkly different dentition.

In their paper identifying *Ontocetus emmonsi* as an extinct walrus, Kohno and Ray [38] transferred *Ontocetus oxymycterus* to the fossil sperm whale genus *Scaldicetus*, in line with Kellogg’s [28] favorable comparison. However, *Scaldicetus* is a form taxon that is recognized from many different taxa possessing skull fragments with isolated teeth showing the hallmark apical caps of rugose enamel with gibbous roots. The inferred upper and known lower dentition of *Albicetus* is broadly similar to other physeteroid taxa possessing dentition of the *Scaldicetus* morphotype. For example, a fossil sperm whale from the Miocene of Japan originally named *Scaldicetus shigensis* Hirota and Barnes [65] represents one of the more complete physeteroids possessing dentition of the *Scaldicetus* morphotype. Kimura et al. [4] later redescribed this specimen and moved it to a new genus *Brygmophyseter* (this latter genus name has priority over *Naganocetus* Bianucci and Landini [22], which was published one month after Kimura et al. [4], despite being accepted for publication prior to the submission of the manuscript by Kimura et al. [4]). *Albicetus* differs from *Brygmophyseter* in having premaxillae that gradually expand posteriorly and lacking laterally crested maxillae that incline medially on the anterior portion of the rostrum to form a continuous surface with the premaxillae. Instead, the maxillae on *Albicetus* incline dorsomedially to meet the premaxillae, which then are convex towards the midline. Although the premaxillae in *Brygmophyseter* closely appress and roof over the mesorostral groove in the anterior portion of the rostrum, the premaxillae are not fused medially.

None of the diagnostic features used to distinguish *Albicetus* as a new genus (such as the roofed over mesorostral groove, presence of upper teeth and enameled tooth caps) are unique to the genus. However, it is the specific combination of these features seen in *Albicetus* that sets it apart from any of the other fossil physeteroid genera. In comparison to other stem and crown Physeteroidea, *Albicetus* is most comparable in size only to *Zygophyseter* and *Brygmophyseter*. It is smaller than *Physeter* and *Livyatan*, but larger than all living and fossil pan-kogiids (sensu [9]), and fossil physeteroids such as *Acrophyseter*, *Orycterocetus* and stem-ward taxa. *Albicetus* differs from *Livyatan* and *Physeter* in lacking an elongated supracranial basin on the rostrum [5]. The premaxillae of *Albicetus* are wider than the maxillae at points in dorsal view, differing it from *Placoziphius* Van Beneden [66–67]. *Albicetus* differs from *Physeterula* Van Beneden 1877 [68] in having maxillae visible in dorsal view for more than 50% of rostral length, and possessing absolutely (and proportionately) much larger teeth [20]. *Albicetus* differs from *Acrophyseter* in lacking a deep, longitudinal groove on the right premaxilla along the rostrum, and in lacking a less pronounced dorsal curvature of the rostrum and mandibles [21]. *Albicetus* differs from *Zygophyseter* in having a wider rostrum in the anterior portion [22]. Although the dentition of *Albicetus* is known from only fragmentary teeth, it is overall similar in general morphology with *Livyatan*, yet *Albicetus* differs from *Livyatan* in having the distal end of the rostrum delineated only by the premaxillae, and having a rostrum laterally concave along the anterior portion [5]. In comparison to *Orycterocetus*, *Albicetus* differs in its retention of enamel, the lack of constriction of more than half of the rostrum anteriorly, and a roofing over of the mesorostral groove [69]. *Albicetus* differs from all Physeteroidea except *Aulophyseter*, *Brygmophyseter*, and *Scaphokogia* in having a mesorostral groove roofed over by the premaxillae [20].

The fossil pan-physeteroids represented in Lambert et al. [5]'s study, which formed the basis for our phylogenetic analysis of *Albicetus*, are almost entirely Northern Hemisphere taxa. This sampling is largely a consequence of historiography, attributable to the prolonged interest in and study of fossil sperm whales near the major centers of learning in Europe, the United States, and Japan. By contrast, there are several South American pan-physeteroids from early Neogene strata that are worthy of renewed study, especially *Idiorophus patagonicus* (Kellogg [28]), *Diaphorocetus poucheti* (Ameghino [70]) and '*Aulophyseter*' *rionegrensis* (Gondar [71]), among others. Based on existing descriptions, *Idiorophus* and *Diaphorocetus* appear to represent pan-physeteroids with small-toothed and relatively slender rostral morphologies, similar to *Orycterocetus* in the Northern Hemisphere and very different from *Albicetus*. Cozzuol [72] referred '*Aulophyseter*' *rionegrensis* to a new genus in an unpublished dissertation, suggesting that the complete skull upon which this species is based does not represent *Aulophyseter*. The specimen has teeth of a much smaller diameter in comparison to *Albicetus*.

### 3. Taphonomy

Given the bathyal paleodepth for the depositional environment represented in the lower calcareous unit of the Monterey Formation in the Santa Barbara Coastal Plain, it is reasonable to conclude that the source whale for the type specimen of *Albicetus* died at sea, and sank to the seafloor in a distal shelf setting. Such deep-sea environments are where the majority of whalefalls have been discovered since 1977 [73]. Also, the Monterey Formation has produced fossil whalefalls elsewhere in California [74], in similarly bathyal depositional environments as the inferred type locality for *Albicetus*. While there is no evidence of bioerosion, bioencrustation or an associated invertebrate fauna with the type specimen of Albicetus, it is not entirely clear whether such a community might be expected for a physeteroid whalefall, even at the presumed paleodepth for the type specimen's depositional environmental, since reported modern sperm whalefalls derive from experimental, emplaced carcasses [75].

In his initial description, Kellogg [26] argued that the type specimen of *Albicetus* likely decayed substantially prior to burial, resulting in tooth crowns broken off from teeth still in their alveoli and upper teeth that “[dropped] out of the alveoli in the upper jaws after the skull was covered with sediments as several were found in the matrix.” Although there are no documented modern analogs in cetaceans, this pathway of decay is entirely feasible, given the weight of the individual teeth. While there are no data recorded by Roe or Kellogg about the articulation of the postcranium in the type specimen, the clean break of the proximal end of the rostrum suggests that the skull had been intact before burial. This contention is also supported by the articulation of both mandibles to the skull in near-life position. It is likely that the source carcass, or at least the cranium and mandibles, reached the seafloor in articulation. It also appears that the weight of the rostrum provided sufficient force to displace the mandibles prior to burial.

It is unknown whether more of the skeleton of *Albicetus* still remains in the sea cliffs at the type locality near the original Santa Barbara Lighthouse. Regardless, it is almost certain that any remaining skeletal material would have been eroded away, now over a century later. Moreover, the lack of specific locality information makes it impossible to relocate the exact spot from which the specimen was extracted. If the skeleton remained relatively well articulated in the cliff face, it would suggest that the observed preservation arose from one or more factors, including rapid burial, quiet conditions and/or limited scavenging [26].

### 4. Body size & phylogeny

Kellogg [26] provided an initial estimate of 4 to 5 meters for the length of the complete skull of *Albicetus*, perhaps based mostly on general appearances, which was closer to our calculation for the full body length rather than the condylobasal length. Based on crania from other fossil physeteroids, we judged our estimated reconstructed condylobasal length as relatively robust, although the discovery of more complete material will surely provided a better basis for this evaluation. Our estimated total length for *Albicetus* (TL = 5.9–6.3 m) is smaller than *Livyatan* and *Physeter*, but within the size range for other stem physeteroids such as *Brygmophyseter*.

Lindberg and Pyenson [76] argued that the evolution of echolocation in odontocetes was an ecological adaptation for pursuing abundant pelagic prey at depth, such as diel-migrating cephalopods. Under this evolutionary scenario, the innovation of echolocation was an escalation response to deep-diving prey behaviour, culminating in an evolutionary arms race of body size increases for both the deepest diving odontocetes and their prey, which include the largest known cephalopods [77]. Large body size appears to confer several selective benefits for deep-diving in marine mammals [78], although there are likely other physiological and even molecular mechanisms underlying this convergent trait [79]. Given this hypothesis, we would expect to see phylogenetic trends towards increasing body size to extant *Physeter*, although our findings (Fig 15) show a different pattern.

First, large body size in physeteroids (i.e., total lengths >6 m) was achieved by the mid Miocene, and among different lineages (e.g., *Albicetus*, *Brygmophyseter*, *Livyatan*) in the Langhian and Serravallian, and then again in the late Miocene with *Zygophyseter* and *Physeterula* [68]. No fossil or extant kogiids attained such sizes, and remained generally within the same size range for their entire clade history, despite morphological changes to their supracranial basin [9]. Among fossil physeteroids with large body size, all show both functional upper and lower dentition. *Aulophyseter*, which is smaller than all of the aforementioned taxa, does not have upper alveoli, and it is the sister taxon to extant *Physeter*, which is the largest physeteroid ever, and similarly lacks functional upper dentition. The occurrence of functional upper and lower dentition in many of the largest fossil physeteroids led Lambert et al. [5] to suggest that hypercarnivory (and in particular, predation of marine mammals) was a primary driver for large body size in these lineages, as opposed to deep diving. This hypothesis specifically posits that the middle Miocene provided a peak in richness for marine mammals [25], the presumed prey items. *Albicetus* fits the pattern of this hypothesis, as a large pan-physeteroid with functional upper and lower dentition from the middle Miocene. However, beyond qualitative characterization of feeding morphology, more data (e.g., isotopic analyses of physeteroid tooth enamel) would provide better support for this contention.

Nonetheless, the co-occurrence of multiple large, putatively hypercarnivorous physeteroids in the middle Miocene, as opposed to the singular teuthophagous *Physeter* alive today (with comparatively small *Kogia* spp.), points to unusual structuring in Miocene marine communities that have no analogs in today’s oceans, where hypercarnivory is rare [80]. Also, the unusual composition of mid Miocene physeteroid communities provides yet another instance in the marine mammal fossil record where extant diversity provides a poor guide for clade history, and vice versa [81].

Our trait mapping of body size (using condylobasal length) on our consensus tree also revealed that most pan-physeteroids were larger than pan-kogiids, although *Livyatan* and *Physeter* represent two independent excursions, at separate geological times, to extremely large cranial size (and overall body size) (Fig 15). *Albicetus*, by comparison, falls within the size range of *Brygmophyseter*, *Aulophyseter* and other large-size fossil physeteroids. As previously mentioned, the Langhian appears to represent a time of unusual richness in stem physeteroids that overlapped in stratigraphic range within about 3 million years, and, in some cases, shared ocean basins (e.g., *Brygmophyseter*, *Aulophyseter*, and *Albicetus* all in the North Pacific Ocean). Given their similar body sizes, future investigations might examine how stem physeteroids with putative ecological overlap might have partitioned their resources, in a similar fashion to the ecomorphological and body size structuring of fossil sirenian assemblages in the Cenozoic [81] and, potentially, stem Mysticeti in the Oligocene [82].

## Conclusions

We provided new information about an enigmatic fossil sperm whale from the Miocene of California, *Albicetus oxymycterus*, for which we provided a new genus name because of taxonomic priority of its original name with a fossil walrus. Our redescription of the type specimen of *Albicetus* provides new morphological details, along with revisions to the stratigraphy and locality data, as can best be ascertained given the available historical information. We provided a phylogenetic analysis to determine the relationship of *Albicetus* to other fossil sperm whales, along with body size estimates. Our results indicate that *Albicetus* was a large, stem physeteroid with a seemingly unique combination of diagnostic features observed in no other living or fossil physeteroid.

## Supporting Information

S1 Fig Phylogenetic tree with bootstrap values for Physeteroidea. (TIF)

S2 Fig Measurement diagram. Diagram of measurements taken from the main rostral section and isolated upper rostral fragment from the holotype of *Albicetus oxymycterus* (USNM 10923).
(TIF)

S1 Table Specimens Observed. Fossil marine mammal specimens observed during the writing of this publication.
(DOCX)

S2 Table Matrix constructed in Mesquite for Physeteroidea including *Albicetus*. 0, primitive state; 1, 2, 3, derived states; 0/1, a variable between 0 and 1; 1/2 a variable between 1 and 2; 1/3, a variable between 1 and 3;?, missing character or taxon not coded for this character.
(TIF)

S3 Table Character state descriptions. Following Velez-Juarbe et al. [25]
(DOCX)

S4 Table Table of condylobasal lengths of the Physeteroidea taxa. All approximated (~) measurements are estimates based on fragmentary material. For, *Agorophius*, *Thalassocetus and Praekogia* (denoted by *), estimated CBL were collected from cetacean genera of similar sizes (*Simocetus* for *Agorophius*, *Kogia breviceps* for *Thalassocetus*, and *Praekogia* for *Nanokogia*). Each substituted taxon, in these cases, shares similar skull proportions (e.g., bizygomatic width), even if there are differences in rostral morphology, or other features. Therefore, we think such proxy taxa are justified, given that they do not alter the ultimate size bin in which these taxa belong.
(DOCX)

## Abbreviations

USNM: Department of Paleobiology, National Museum of Natural History, Smithsonian Institution, Washington, District of Columbia, U.S.A.
LACM: Natural History Museum of Los Angeles County, Los Angeles, California, U.S.A.
BE-BVM: Bob Ernst collection, Buena Vista Museum of Natural History, Bakersfield, California, U.S.A.

## References

[1] Linnaeus C. Systema naturae per regna tria naturae, secundum classes, ordines, genera, species, cum characteribus, differentiis, synonymis, locis Tomus 1, Editio decima, reformata. Stockholm: Laurentii Salvii; 1758.

[2] de Blainville H. Sur les cachalots. Ann Fr Étrang d’Anat Physiol. 1838; 2: 335–337.

[3] Owen R. On some Indian Cetacea collected by Walter Elliot, Esq. Trans Zool Soc London. 1866; 6:17–47.

[4] Kimura T, Hasegawa Y, Barnes LG. Fossil sperm whales (Cetacea, Physeteridae) from Gunma and Ibaraki prefectures, Japan; with observations on the Miocene fossil sperm whale *Scaldicetus shigensis* . Bulletin of Gunma Museum of Natural History. 2006;10: 1–23.

[5] Lambert O, Bianucci G, Post K, de Muizon C, Salas-Gismondi, Urbina M, Reumer J. The giant bite of a new raptorial sperm whale from the Miocene epoch of Peru. Nature. 2010;466: 105–108. 10.1038/nature09067 20596020

[6] Watwood SL, Miller PJ, Johnson M, Madsen PT, Tyack PL. Deep-diving foraging behavior of sperm whales (*Physeter macrocephalus*). J Amin Ecol. 2006;75: 814–825. 10.1111/j.1365-2656.2006.01101.x 16689963

[7] Whitehead H. Sperm Whales: Social Evolution in the Ocean. Chicago: University of Chicago Press; 2003.

[8] Nowak RM. Walker’s Mammals of the World. Baltimore: Johns Hopkins University Press; 1999.

[9] Velez-Juarbe J, Wood AR, De Gracia C, Hendy AJW. Evolutionary patterns among living and fossil kogiid sperm whales: evidence from the Neogene of Central America. PLoS ONE. 2015 4 29 10.1371/journal.pone.0123909 PMC4414568 25923213

[10] Flower WH. On the Osteology of the Cachalot or Sperm Whale (*Physeter macrocephalus*). Transactions of the Zoological Society of London. 1868;6: 309–372.

[11] Berzin AA. The Sperm Whale. Jerusalem: Israel Program for Scientific Translations; 1972.

[12] Geisler JH, Sanders AE. Morphological evidence for the phylogeny of Cetacea. J Mamm Evol. 2003;10: 23–129.

[13] McGowen MR, Spaulding M, Gatesy J. Divergence date estimation and a comprehensive molecular tree of extant cetaceans. Mol Phylogenet Evol. 2009;53: 891–906. 10.1016/j.ympev.2009.08.018 19699809

[14] Gatesy J, Geisler JH, Chang J, Buell C, Berta A, Meredith RW, et al A phylogenetic blueprint for a modern whale. Mol Phylogenet Evol. 2013;66: 479–506. 10.1016/j.ympev.2012.10.012 23103570

[15] Geisler JH, McGowen MR, Yang G, Gatesy J. A supermatrix analysis of genomic, morphological, and paleontological data from crown Cetacea. BMC Evol Biol. 2011;11: 112 10.1186/1471-2148-11-112 21518443 PMC3114740

[16] Heyning JE. Sperm whale phylogeny revisited: analysis of the morphological evidence. Mar Mamm Sci. 1997;13: 596–613.

[17] Messenger SL, McGuire JA. Morphology, molecules, and the phylogenetics of cetaceans. Systematic Biology. 1998;47: 90–124. 12064244 10.1080/106351598261058

[18] Mchedlidze GA. Nekotorye obshchie cherty istorii kitoobraznykh Vitaliano DB, transl. Tbilisi: Metsniereba; 1970.

[19] Barnes LG, Domning DP, Ray CE. Status of studies on fossil marine mammals. Mar Mamm Sci. 1985;1: 15–53.

[20] Lambert O. Sperm whales from the Miocene of the North Sea: a re-appraisal. Sciences de la Terre. 2008;78: 277–316.

[21] Lambert O, Bianucci G, de Muizon C. A new stem-sperm whale (Cetacea, Odontoceti, Physeteroidea) from the Latest Miocene of Peru. Comptes Rendus Palevol. 2008;7: 361–369.

[22] Bianucci G, Landini W. Killer sperm whale: a new basal physeteroid (Mammalia, Cetacea) from the Late Miocene of Italy. Zoo J Linn Soc. 2006;14: 103–131.

[23] Hampe O. Middle/late Miocene hoplocetine sperm whale remains (Odontoceti: Physeteridae) of North Germany with an emended classification of the Hoplocetinae. Fossil Record. 2006;9: 61–86.

[24] Fitzgerald EMG. A fossil sperm whale (Cetacea, Physeteroidea) from the Pleistocene of Nauru, Equatorial Southwest Pacific. Journal of Vertebrate Paleontology. 2011;31: 929–931.

[25] Kellogg R. Study of the skull of a fossil sperm whale from the Temblor Miocene of Southern California In: Kellogg R, Merriam JC, Stock C, Chaney RW, Mason HL. Additions to the paleontology of the Pacific coast and Great Basin regions of North America. Washington: Carnegie Institution of Washington; 1927 pp. 3–22.

[26] Fordyce RE, Muizon C. Evolutionary history of whales: a review In: Mazin JM, de Buffrenil V, editors. Secondary adaptation of tetrapods to life in water. Munich: Verlag Friedrich Pfeil; 2001.

[27] Uhen MD, Pyenson ND. Diversity estimates, biases, and historiographic effects: resolving cetacean diversity in the Tertiary. Palaeontol Electronica. 2007:10; 11A–22.

[28] Kellogg R. A fossil physeteroid cetacean from Santa Barbara County, California In: The Proceedings of the United States National Museum, vol.66 Washington: G. P. O; 1925.

[29] Leidy J. Remarks on *Dromatherium sylvestre* and *Ontocetus emmonsi* . Proceedings of the Academy of Natural Sciences of Philadelphia. 1860;11: 162.

[30] Leidy J. The extinct mammalian fauna of Dakota and Nebraska, including an account of some allied forms from other localities, together with a synopsis of the mammalian remains of North America Philadelphia: Journal of the Academy of Natural Sciences; 1869: 440.

[31] Emmons E. Manual of geology: designed for the use of colleges and academies New York: A.S. Barnes and Burr; 1860.

[32] Brandt JF. Untersuchungen über die fossilen und subfossilen Cetaceen Europa's. Mémoires de l'Académie Impériale des Sciences de Saint-Petersbourg, Series 7. 1873;20:1–372.

[33] Spamer EE, Daeschler E, and Vostreys-Shapiro LG. A study of fossil vertebrate types in the Academy of Natural Sciences of Philadelphia. Academy of Natural Sciences of Philadelphia Special Publication. 1995;16: 1–434.

[34] Matsumoto H. On some fossil Cetaceans of Japan. Science Reports of the Tokyo Imperial University. 1926;10: 17–27.

[35] Shikama T, Hasegawa Y, Otsuka H. Geological range of mammals in the Japanese Neogene. Memoirs of the Geological Society of Japan. 1973;8: 137–141.

[36] Okazaki Y. Miocene long-snouted porpoises from the Mizunami Group, Central Japan. Bulletin of the Mizunami Fossil Museum. 1976;3: 25–39.

[37] Ray CE. The relationships of *Hemicaulodon effodiens* Cope 1869 (Mammalia: Odobenidae). Proceedings of the Biological Society of Washington. 1975;88: 281–304.

[38] Kohno N, Ray CE. Pliocene walruses from the Yorktown Formation of Virginia and North Carolina, and a systematic revision of the North Atlantic Pliocene walruses. Virginia Museum of Natural History Special Publication. 2008;14: 39–80.

[39] Boessenecker RW, Churchill M. A reevaluation of the morphology, paleoecology, and phylogenetic relationships of the enigmatic walrus *Pelagiarctos* . PLoS ONE. 2013 10.1371/journal.pone.0054311 PMC3546998 23342129

[40] Churchill M, Clementz MT, Kohno N. Cope's rule and the evolution of body size in Pinnipedimorpha (Mammalia: Carnivora). Evolution. 2015;69: 201–215. 10.1111/evo.12560 25355195

[41] Du Bus BAL. Sur quelques Mammifères du Crag d’Anvers. Bulletin de l'Académie Royale des Sciences, des Lettres et des Beaux-Arts de Belgique. 1867;24: 562–577.

[42] Mead JG, Fordyce RE. The Therian Skull: a lexicon with emphasis on the odontocetes Washington: Smithsonian Contributions to Zoology; 2009.

[43] Pyenson ND, Sponberg SN. Reconstructing body size in extinct crown Cetacea (Neoceti) using allometry, phylogenetic methods and tests from the fossil record. J Mamm Evol. 2011;18: 269–288.

[44] Cope ED. An addition to the vertebrate fauna of the Miocene period, with a synopsis of the extinct Cetacea of the United States. Proceedings of the Academy of Natural Sciences of Philadelphia. 1867;19: 138–157.

[45] Muizon C. Les vertebres fossiles de la Formation Pisco (Perou), Troisieme partie: Les Odontocetes (Cetacea, Mammalia) du Miocene. France: Editions Recherche sur les Civilisations; 1988.

[46] Swofford DL. PAUP* Phylogenetic Analysis Using Parsimony (*and other methods) Version 4. Massachusetts: Sinauer Associates; 2002.

[47] Brisson MJ. Regnum animale in Classes IX distributum, sive synopsis methodica sistens generalem animalium distributionem in Classes IX, et duarum primarum Classium, Quadrupedum scilicet & Cetaceorum, particulare divisionem in Ordines, Sectiones, Genera, et Species Paris: T. Haak; 1762.

[48] Flower WH. Description of the skeleton of Inia geoffrensis and of the skull of Pontoporia blainvillii, with remarks on the systematic position on these animals in the order Cetacea. Transactions of the Zoological Society of London. 1867;6: 87–116.

[49] Gray JE. Synopsis of the species of whales and dolphins in the Collection of the British Museum London: B. Quaritch; 1868.

[50] Melville H. Moby-Dick; or, The Whale. New York City: Bantam Classics; 1981.

[51] Santa Barbara County Genealogical Society. Cemetery Records. 2015. Available: http://sbgen.org/cemeteryRecords.php?page=410&lv=R&sortCol=dya&srch=&cid.

[52] Hope Ranch Home Owners Association. History. 2015. Available: https://www.hoperanch.org/pages/History.htm.

[53] Rowlett R. Lighthouses of the United States: Southern California. The Lighthouse Directory. 2005; 25 Available: https://www.unc.edu/~rowlett/lighthouse/ca2.htm.

[54] United States Coast Guard. Light List Volume VI, Pacific Coast and outlying Pacific Islands. Washington D.C.: G.P.O; 2015.

[55] Minor SA, Kellogg KS, Stanley RG, Gurrola LD, Keller EA, Brandt TR. Geologic Map of the Santa Barbara Coastal Plain Area, Santa Barbara County, California. U.S. Geological Survey Scientific Investigations. 2009. Map 3001, scale 1:25,000, 1 sheet, pamphlet, 38 p.

[56] Kleinpell RM. Miocene stratigraphy of California Oklahoma: American Association of Petroleum Geologists; 1938.

[57] Kleinpell RM. History of stratigraphic paleontology of west coast Tertiary In: Kleinpell RM. The Miocene stratigraphy of California revisited. Oklahoma: American Association of Petroleum Geologists, Studies in Geology no 11; 1980.

[58] Behrensmeyer AK. Taphonomic and Ecological Information from Bone Weathering. Paleobiology. 1978;4: 150–162.

[59] Melnikov VV. The arterial system of the sperm whale (*Physeter macrocephalus)* . J Morphol. 1997;234: 37–50. 9329202 10.1002/(SICI)1097-4687(199710)234:1<37::AID-JMOR4>3.0.CO;2-K

[60] Kellogg R. Two fossil physeteroid whales from California. Contr. Paleontol. 1925;348: 1–34.

[61] Lambert O, Bianucci G, Beatty BL. Bony outgrowths on the jaws of an extinct sperm whale support macroraptorial feeding in several stem physeteroids. Naturewissenchaften. 2014;101: 517–521. 10.1007/s00114-014-1182-2 24821119

[62] Gibbs NJ, Kirk EJ. Erupted upper teeth in a male sperm whale, *Physeter macrocephalus* . N Z J Mar Freshwater Res. 2011;35: 325–327.

[63] Maddison WP, Maddison DR. 2015. Mesquite: A modular system for evolutionary analysis. Version 3.02. Available: http://mesquiteproject.org.

[64] Cope ED. Fourth contribution to the marine fauna of the Miocene Period of the United States. Proceedings of the American Philosphical Society. 1895;34: 135–155.

[65] Hirota K, Barnes LG. A new species of Middle Miocene sperm whale of the genus *Scaldicetus* (Cetacea; Physeteridae) from Shinga-mura, Japan. Island Arc. 1994;3: 453–472.

[66] Van Beneden PJ. Sur un nouveau genre de ziphioide fossil (Placoziphius), trouve a Edeghem, pres d'Anvers. Memoires de L'Academie Royale des Sciences, des Lettres et des Beaux-arts de Belgique. 1869;28: 2–12.

[67] Kazár E. Revised phylogeny of the Physeteridae (Mammalia: Cetacea) in the light of *Placoziphius* VAN BENEDEN, 1869 and *Aulophyseter* KELLOGG, 1927. Bulletin de l’Institut des Sciences Naturelles de Belgique, Sciences de la Terre. 2002;72: 151–170.

[68] Van Beneden PJ. Note sur un Cachalot nain du crag d'Anvers, *Physeterula dubussi* . Bulletins de L'académie Royal des Sciences, des Lettres et des Beaux-Arts. 1877;64: 851–856.

[69] Kellogg R. Pt. 2. The Miocene Calvert Sperm Whale *Orycterocetus* In: Kellogg R. Fossil marine mammals from the Miocene Calvert Formation of Maryland and Virginia. Washington: Museum of Natural History, Smithsonian Instutution; 1965 pp. 47–63.

[70] Ameghino F. Enumeration synoptique des especes de mammifères fossiles des formations éocènes de Patagonie. Boletin de la Academia Nacional de Ciencias en Cordoba (Republica Argentina). 1894;13: 259–452.

[71] Gondar D. La presencia de cetaceos Physeteridae en el Terciario Superior (“Rionegrense”) de la Provincia de Rio Negro. Actas de Primer Congreso Argentino de Paleontologia y Bioestratigrafia. 1974;2: 349–356.

[72] Perez LM, Cione AL, Cozzuol M, Varela AN. A sperm whale (Cetacea: Physeteroidea) from the Paraná Formation (Late Miocene) of Entre Ríos, Argentina. Environment and Taphonomy. Ameghiniana;2011: 648–654.

[73] Smith CR, Glover AG, Treude T, Higgs ND, Amon DJ. Whale-Fall Ecosystems: Recent Insights into Ecology, Paleoecology, and Evolution. Ann Rev Mar Sci. 2015;7: 571–596. 10.1146/annurev-marine-010213-135144 25251277

[74] Pyenson ND, Haasl DM. Miocene whale-fall from California demonstrates that cetacean size did not determine the evolution of modern whale-fall communities. Biology Letters. 2007;3: 709–711. 17848361 10.1098/rsbl.2007.0342 PMC2391211

[75] Fugiwara Y, Kawato M, Yamamoto T, Yamanaka T, Sato-Okoshi W, Noda Chiyako, et al Three-year investigations into sperm whale-fall ecosystems in Japan. Marine Ecology. 2007;28: 219–232.

[76] Lindberg DR, Pyenson ND. Things that go bump in the night: evolutionary interactions between cephalopods and cetaceans in the tertiary. Lethaia. 2007;40: 335–343.

[77] Clarke MR. Cephalopods as prey. III. Cetaceans. Philos Trans R Soc Lond B Biol Scie. 1996;351: 1053–1065.

[78] Williams TM, Davis RW, Fuiman LA, Francis J, Le BJ, Horning M, et al Sink or swim: strategies for cost-efficient diving by marine mammals. 2000;288: 133–136. 10753116 10.1126/science.288.5463.133

[79] Mirceta S, Signore AV, Burns JM, Cossins AR, Campbell KL, Berenbrink M. Evolution of mammalian diving capacity traced by myoglobin net surface charge. Science. 2013 10.1126/science.1234192 23766330

[80] Kelley NP, Pyenson ND. Evolutionary innovation and ecology in marine tetrapods from the Triassic to the Anthropocene. Science. 2015. doi: 0.1126/science.aaa3716 10.1126/science.aaa3716 25883362

[81] Velez-Juarbe J, Domning DP, Pyenson ND. Iterative Evolution of Sympatric Seacow (Dugongidae, Sirenia) Assemblages during the Past ~26 Million Years. PLoS ONE. 2012 10.1371/journal.pone.0031294 PMC3272043 22319622

[82] Tsai CH, Ando T. Niche partitioning in Oligocene toothed mysticetes (Mysticeti: Aetiocetidae). J Mamm Evol. 2015 10.1007/s10914-015-9292-y

