# Supplementary figures and images for: Albicetus oxymycterus, a New Generic Name and Redescription of a Basal Physeteroid (Mammalia, Cetacea) from the Miocene of California, and the Evolution of Body Size in Sperm Whales
Source: PLoS One. 2015 Dec 9;10(12):e0135551. doi: 10.1371/journal.pone.0135551 (PMC4674121; doi:10.1371/journal.pone.0135551)

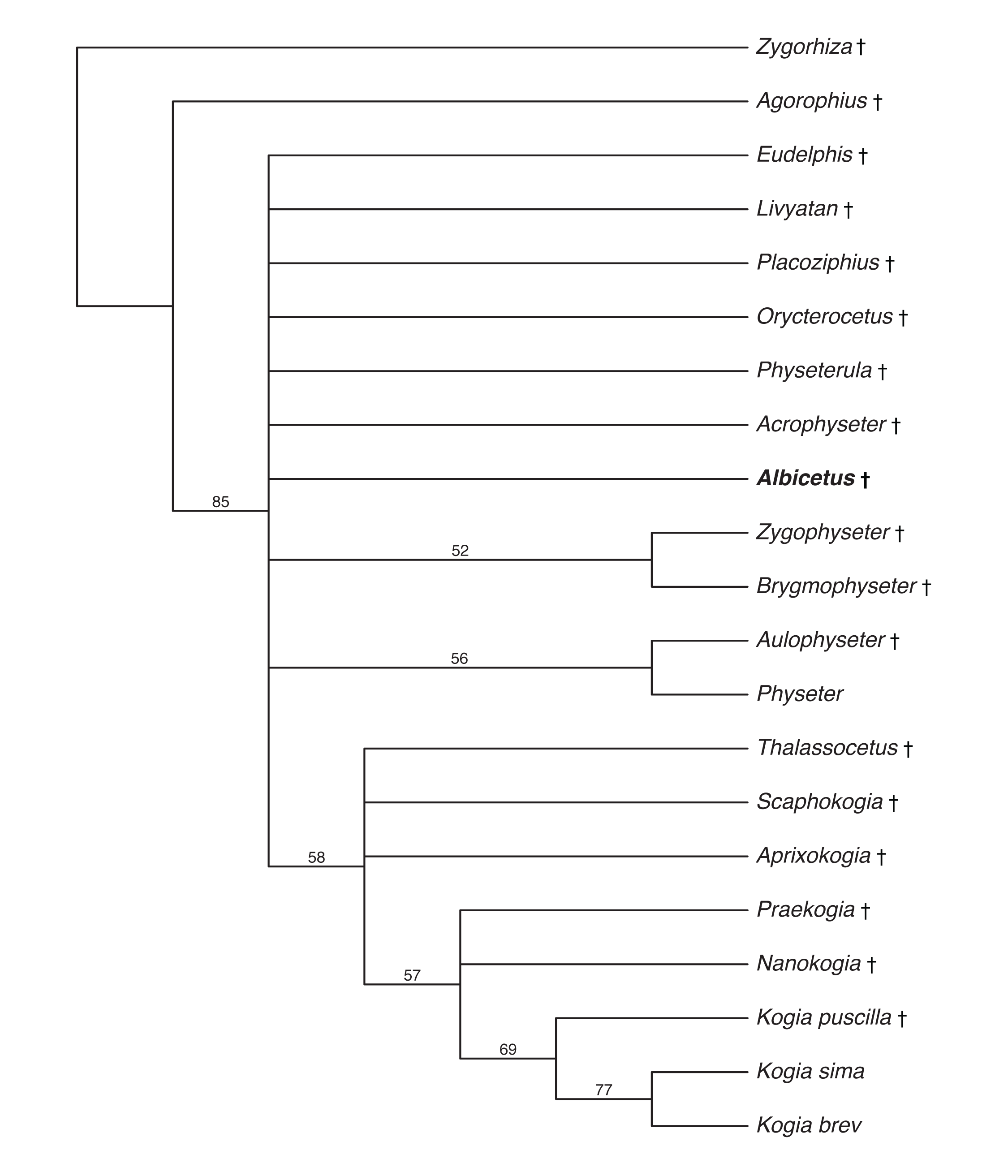

Supplement: S1 Fig — (TIF) [file pone.0135551.s001.tif]

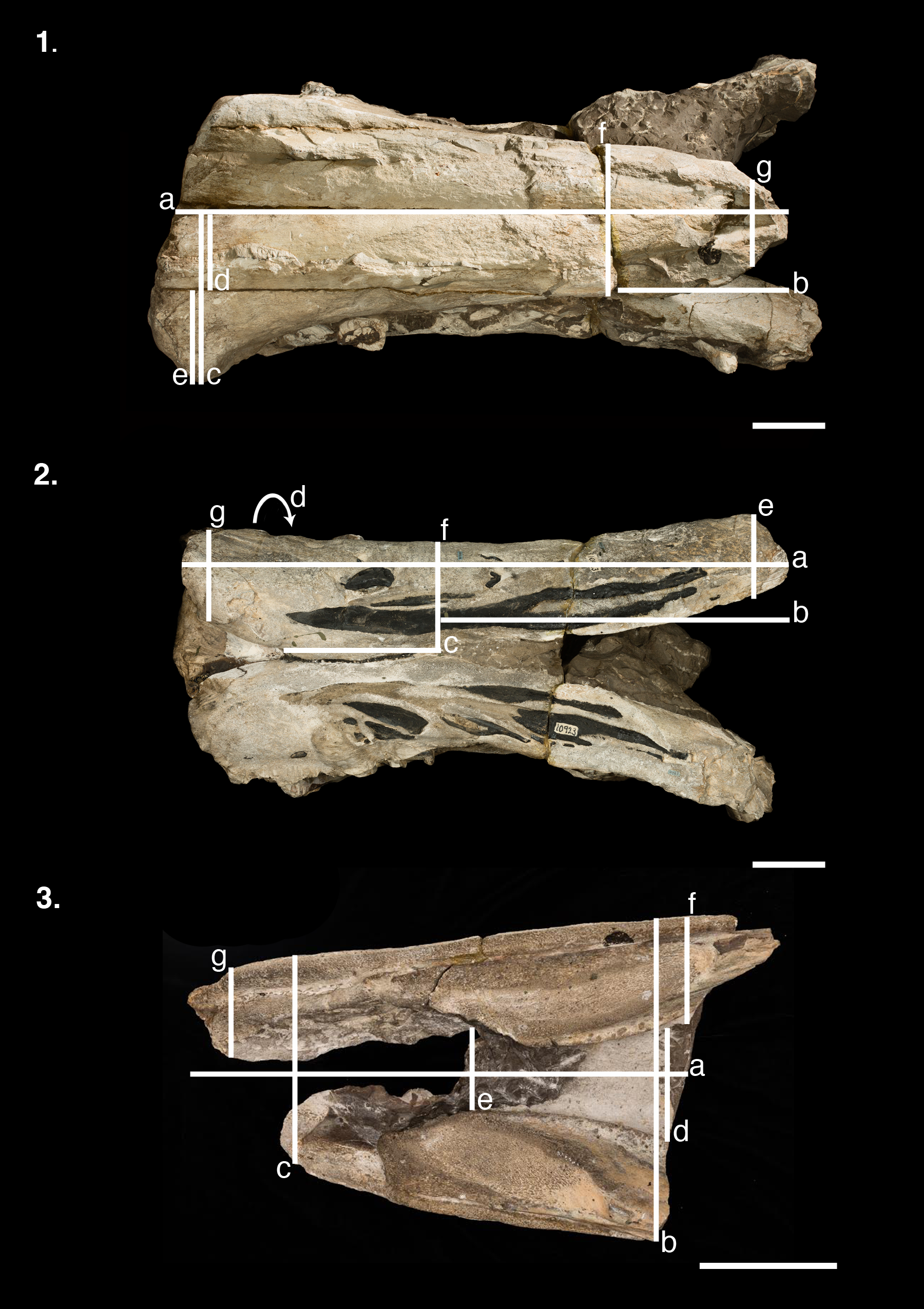

Supplement: S2 Fig — Diagram of measurements taken from the main rostral section and isolated upper rostral fragment from the holotype of Albicetus oxymycterus (USNM 10923). (TIF) [file pone.0135551.s002.tif]

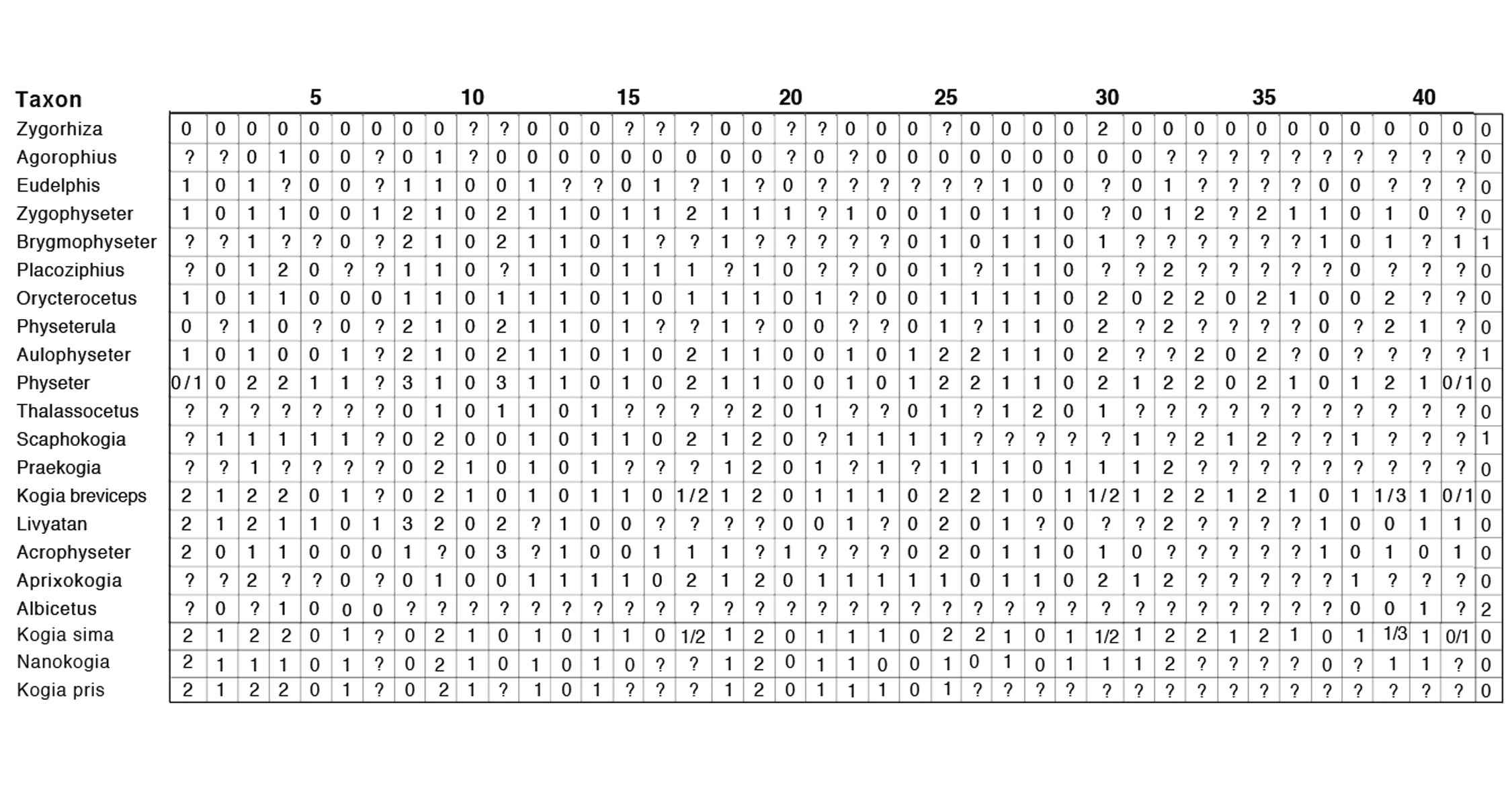

Supplement: S2 Table — 0, primitive state; 1, 2, 3, derived states; 0/1, a variable between 0 and 1; 1/2 a variable between 1 and 2; 1/3, a variable between 1 and 3;?, missing character or taxon not coded for this character. (TIF) [file pone.0135551.s004.tif]
