# Supplementary material for: Albicetus oxymycterus, a New Generic Name and Redescription of a Basal Physeteroid (Mammalia, Cetacea) from the Miocene of California, and the Evolution of Body Size in Sperm Whales
Source: PLoS One. 2015 Dec 9;10(12):e0135551. doi: 10.1371/journal.pone.0135551 (PMC4674121; doi:10.1371/journal.pone.0135551)
Supplement: S1 Table — Fossil marine mammal specimens observed during the writing of this publication. (DOCX) [file pone.0135551.s003.docx]

| **Species** | **Specimens** |
| --- | --- |
| *Physeter macrocephalus* | USNM 253051  USNM 395398  USNM 35315  USNM 301634  USNM 15274  USNM 239284  USNM 239337  USNM 550876 |
| *Aulophyseter morricei* | USNM 11230 |
| *Orycterocetus crocodilinus* | USNM 22926  USNM 14730  USNM 14729  USNM 22931 |
| *Orycterocetus mediatlanticus* | USNM 9463 |
| *Ontocetus emmonsi* | USNM 329064 |

*Information on other taxa used in the morphological comparisons was collected from published literature.
