# Supplementary material for: Albicetus oxymycterus, a New Generic Name and Redescription of a Basal Physeteroid (Mammalia, Cetacea) from the Miocene of California, and the Evolution of Body Size in Sperm Whales
Source: PLoS One. 2015 Dec 9;10(12):e0135551. doi: 10.1371/journal.pone.0135551 (PMC4674121; doi:10.1371/journal.pone.0135551)
Supplement: S3 Table — Following Velez-Juarbe et al. [25] (DOCX) [file pone.0135551.s005.docx]

|  | **Character** | **State 0 (Primitive state)** | **State 1 (Derived state)** | **State 2 (Derived state)** | **State 3 (Derived state)** |
| --- | --- | --- | --- | --- | --- |
| 1 | Rostrum Length | Rostrum elongated, ratio between rostrum length and skull length | Ratio <- 1.2 and -> 0.95 | Short rostrum, ratio < 0.95 |  |
| 2 | Maxillae, premaxillae and vomer, all reaching the tip of the rostrum which is formed only by the premaxillae | Absent | Present |  |  |
| 3 | Supracranial basin of the skull | Absent | Present | Extended onto the whole dorsal surface of the rostrum |  |
| 4 | Dorsal exposure of the maxilla on the rostrum | Exposure limited to less than half the rostrum length | Maxilla exposed on more than half the length of the rostrum, narrower than the premaxilla at some levels | Wider than the premaxilla all along |  |
| 5 | Construction of premaxillae anterior to antorbital notch followed by anterior expansion | Absent, maxilla-premaxilla suture on the rostrum roughly anteriorly directed | Present, maxilla-premaxilla suture distinctly anteriolaterally directed |  |  |
| 6 | Upper tooth row | Deep alveoli | Alveoli shallow or absent |  |  |
| 7 | Premaxillary Teeth | Present | Absent |  |  |
| 8 | Maximum width of skull (postorbital or bizygomatic width) | < 40 cm | ->40 and < 60 cm | < 100 cm | -> 100 cm |
| 9 | Antorbital notch | Absent | Present | Transformed into a very narrow slit |  |
| 10 | Antorbital notch | Outside the supracranial basin | Inside the supracranial basin |  |  |
| 11 | Number and size of dorsal infraorbital foramina, in the area of the right antorbital notch and posteriorly | Small to moderate size foramina, at least 3-4 | Three large foramina | Two large foramina | One large foramen (max. incisure) |
| 12 | Right premaxilla | Posteriorly extended as the left premaxilla | More posteriorly endtended than the left premaxilla |  |  |
| 13 | Right premaxilla | Not widened posteriorly | Posterior extremity of the right premaxilla laterally widened, occupying at least one third of the width of the supracranial basin |  |  |
| 14 | Presence of a sagittal crest | Absent | Present as a shelf covered by the pointed right premaxilla |  |  |
| 15 | Left premaxillary foramen very small or absent | Absent | Present |  |  |
| 16 | Increase in size of right premaxillary foramen | Absent, ratio between width of foramen and width of premaxilla at <- 0.20 | Present, ratio > 0.20 |  |  |
| 17 | Anteroposterior level of right premaxillary foramen | Distinctly anterior to antorbital notch | Slightly anterior to antorbital notch | Same level or posterior to antorbital notch |  |
| 18 | Asymmetry of bony nares | Absent or reduced | Strong, left bony naris significantly larger than right naris |  |  |
| 19 | Lack of nasals | Both nasal present | One nasal absent | Both nasal absent |  |
| 20 | Widening of the supracranial basin on the right side | Absent | Present, basin overhangs the right orbit |  |  |
| 21 | Right maxilla reaching the sagittal plan of the skull on the posterior wall of the supracranial basin | Absent | Present |  |  |
| 22 | Fusion of lacrimal and jugal | Absent | Present |  |  |
| 23 | Projection of the lacrimal-jugal between frontal and maxilla | Short or absent | Long |  |  |
| 24 | Preorbital process considerably lower than the elevated dorsolateral margin of the rostrum base | Absent | Present |  |  |
| 25 | Fontal-maxilla suture, with skull in lateral view | Forming an angle < 15° from the axis of the rostrum | 15-35° | > 35° |  |
| 26 | Temporal fossa | Anteroposteriorly longer than distance between preorbital proess of maxilla and anterior wall of temporal fossa | Approximately same length | Distinctly shorter |  |
| 27 | Zygomatic process of squamosal in lateral view | “L”- shaped with dorsal margin ventrally bending in its posterior portion | Triangular, with dorsal margin dorsally bending in its posterior portion |  |  |
| 28 | Postglenoid process of the squamosal | Significantly ventrally longer than post-tympanic process | Roughly same ventral extent as post-tympanic process |  |  |
| 29 | In lateral view, wide notch posterior to the postglenoid process of the squamosal for the enlarged posterior process of the tympanic | Absent | Present |  |  |
| 30 | Occipital shield | Convex and forming an angle of about 40° from the axis of the rostrum | As state 0 with ngle of about 60° | Flat or concave forming an angle of about 90° |  |
| 31 | Long axis of the skull | Roughly parallel to the long axis of the body (perpendicular to the surface of the occipital condyles) | Projected ventrally |  |  |
| 32 | Falciform process of the squamosal | Contacting the corresponding pterygoid | Forming a thin plate not contacting the pterygoid | Reduced to a simple peg or absent |  |
| 33 | Anterior bullar facet of the periotic | Very anteroposteriorly elongated | Reduced | Absent or very small |  |
| 34 | Posterior extension of the posterior process of the periotic parallel to the general plane of the bone and not ventrally oriented | Absent | Present |  |  |
| 35 | Accessory ossicle of the tympanic bulla | Absent | Present | Present and partially fused with the anterior process |  |
| 36 | Involucrum of the tympanic bulla with an evident central concavity, visible in ventral and medial views, due to the marked pachyostosis of its anterior and posterior portion | Absent | Present |  |  |
| 37 | Size of teeth (greatest transverse diameter of root expressed as percentage of the maximum width of skull) | < 5% | > 5% |  |  |
| 38 | Loss of dental enamel | Absent | Present |  |  |
| 39 | Number of mandibular teeth | 11 | 12-14 | > 14 |  |
| 40 | Labiolingual compression of the posterior lower teeth (portion out of the alveolus) | Strong | Weak or absent |  |  |
| 41 | Ventral position of the mandibular condyle | Absent, well developed angular process | Present, angular process low or absent |  |  |
| 42 | Mesorostral groove | open | Partially open at the level of the antorbital notch | Closed to the level of the antorbital notches and with the premaxillae angled downward into the midline, creating a trough down the middle of the rostrum |  |
