# Supplementary material for: Albicetus oxymycterus, a New Generic Name and Redescription of a Basal Physeteroid (Mammalia, Cetacea) from the Miocene of California, and the Evolution of Body Size in Sperm Whales
Source: PLoS One. 2015 Dec 9;10(12):e0135551. doi: 10.1371/journal.pone.0135551 (PMC4674121; doi:10.1371/journal.pone.0135551)
Supplement: S4 Table — All approximated (~) measurements are estimates based on fragmentary material. For, Agorophius, Thalassocetus and Praekogia (denoted by *), estimated CBL were collected from cetacean genera of similar sizes (Simocetus for Agorophius, Kogia breviceps for Thalassocetus, and Praekogia for Nanokogia). Each substituted taxon, in these cases, shares similar skull proportions (e.g., bizygomatic width), even if there are differences in rostral morphology, or other features. Therefore, we think such proxy taxa are justified, given that they do not alter the ultimate size bin in which these taxa belong. (DOCX) [file pone.0135551.s006.docx]

| **Taxon** | **Condylobasal Length (cm)** |
| --- | --- |
| *Zygorhiza* | ~73.4 |
| *Agorophius* | *165.0 |
| *Eudelphis* | ~ 95.2 |
| *Zygophyseter* | 148.0 |
| *Brygmophyseter* | ~ 150.0 |
| *Livyatan* | ~ 294.0 |
| *Placoziphius* | ~ 67.0 |
| *Orycterocetus* | 77.3 |
| *Physeterula* | ~ 137.5 |
| *Aulophyseter* | 106.7 |
| *Acrophyseter* | 81.5 |
| *Physeter* | 518.0 |
| *Thalassocetus* | *38.0 |
| *Scaphokogia* | ~ 51.0 |
| *Praekogia* | *34.8 |
| *Kogia sima* | 29.8 |
| *Kogia breviceps* | 47.5 |
| *Kogia puscilla* | 27.0 |
| *Aprixokogia* | 43.0 |
| *Nanokogia* | 34.8 |
| *Albicetus* | ~135.6 |
